# Repositioning linifanib as a potent anti-necroptosis agent for sepsis

**DOI:** 10.1038/s41420-023-01351-y

**Published:** 2023-02-10

**Authors:** Liang Yu, Kai Yang, Xiaoyan He, Min Li, Lin Gao, Yunhong Zha

**Affiliations:** 1 School of Computer Science and Technology, Xidian University, Xi’an, Shaanxi China; 2 Department of Neurology, Institute of Neural Regeneration and Repair, The First Hospital of Yichang, Three Gorges University College of Medicine, Yichang, China

**Keywords:** Virtual screening, Sepsis

## Abstract

Sepsis is a systemic inflammatory syndrome (SIRS) caused by acute microbial infection, and it has an extremely high mortality rate. Tumor necrosis factor-α (TNF-α)-induced necroptosis contributes to the pathophysiology of sepsis, so inhibiting necroptosis might be expected to improve clinical outcomes in septic patients. Here we predicted candidate drugs for treating sepsis in silico by combining genes differentially expressed in septic patients and controls combined with interrogation of the Library of Integrated Network-based Cellular Signatures (LINCS) L1000 perturbation database. Sixteen candidate drugs were screened out through bioinformatics analysis, and the top candidate linifanib was validated in cellular and mouse models of TNF-α-induced necroptosis. Cell viability was measured using a luminescent ATP assay, while the effects of linifanib on necroptosis were investigated by western blotting, immunoprecipitation, and RIPK1 kinase assays. Linifanib effectively protected cells from necroptosis and rescued SIRS mice from TNF-α-induced shock and death. In vitro, linifanib directly suppressed RIPK1 kinase activity. In vivo, linifanib effectively reduced overexpressed IL-6, a marker of sepsis severity, in the lungs of SIRS mice. Our preclinical evidence using an integrated in silico and experimental drug repositioning approach supports the potential clinical utility of linifanib in septic patients. Further clinical validation is now warranted.

## Introduction

Sepsis is a systemic inflammatory syndrome (SIRS) caused by acute microbial infection. It is a serious, life-threatening illness with a 30–50% mortality rate despite antibiotic treatment [1–3]. Given this high morbidity and mortality, the global Surviving Sepsis Campaign was initiated to help improve the treatment of sepsis and septic shock [4], recognizing an acute need for new and more effective therapeutic agents [5].

When sepsis progresses to septic shock, the consequent cytokine “storms” and SIRS injure multiple organs. At least some of the pathobiology of sepsis is driven by necroptosis, usually initiated in a TNF-α-dependent manner, which initiates downstream signaling cascades driving the production of proinflammatory cytokines [6, 7]. Therefore, blocking TNF-α-induced necroptosis might be a useful approach to mitigating massive cytokine release, preventing severe sepsis and, consequently, improving clinical outcomes [8]. However, no inhibitors of necroptosis are currently in clinical use [9].

Here we sought to employ a systematic drug repositioning [10, 11] bioinformatics approach to identify existing FDA-approved drugs that might also be useful to treat sepsis [12]. We propose a new approach to identify therapies to treat sepsis from existing small molecule agents by first investigating differences in gene expression and functional pathways in circulating nucleated cells in patients with and without sepsis. Our approach comprehensively considers genome-wide expression profiles in sepsis and, using pattern matching, identifies drugs that reverse these changes in gene expression. We validate our top candidate drug in a TNF-induced necroptosis model in vitro and the TNF-α-induced SIRS mouse model in vivo. Our study shows that a drug repositioning strategy using bioinformatic predictions combined with experimental validation is a robust way to discover new management strategies for sepsis.

## Results

### Overview of the drug repositioning strategy

**Fig. 1:**
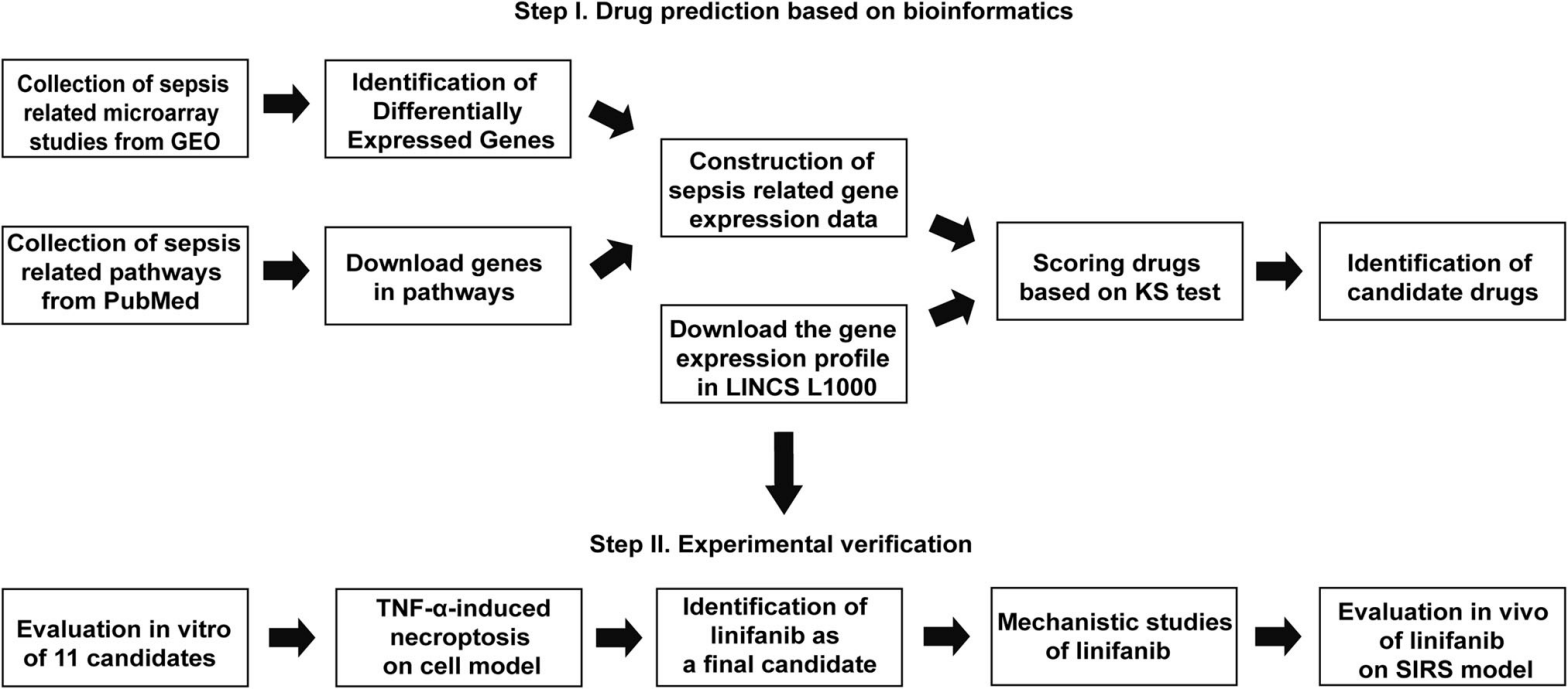
Experimental design. For virtual screening of sepsis drugs, sepsis-related signaling pathways and datasets were retrieved from a public database. DEGs were extracted from sepsis-related microarray data from GEO. Sepsis-related gene expression data and gene expression profiles in the L1000 database were used to calculate therapeutic scores to obtain top candidate compounds. In experimental validation, FDA-approved drug linifanib was shown to have therapeutic effects in cellular and animal models of necroptosis.

Three sepsis-related datasets were identified in the GEO database, which were subjected to bioinformatics analysis to determine differentially expressed genes (DEGs): (i) related to the pathogenesis of sepsis by comparing healthy subjects and patients with sepsis; and (ii) related to patient mortality by comparing survivors and non-survivors of sepsis. Signaling pathways related to sepsis were identified from the literature. Finally, according to the genes expressed in sepsis-related pathways and the induced gene expression profiles in human cell lines treated with small molecules and drugs in the L1000 library, a pattern matching method was designed based on the Kolmogorov–Smirnov test. For each sepsis-related dataset, treatment scores were calculated for all drugs in the L1000 library, and the drugs were sorted. The top 60 drugs in the three datasets were crosschecked in the Comparative Toxicogenomics Database (CTD), and 16 drugs previously unrelated to sepsis or without information in the database were deemed top candidate drugs predicted to treat sepsis (Fig. 1).

### Identification of sepsis-related gene expression profiles

**Fig. 2:**
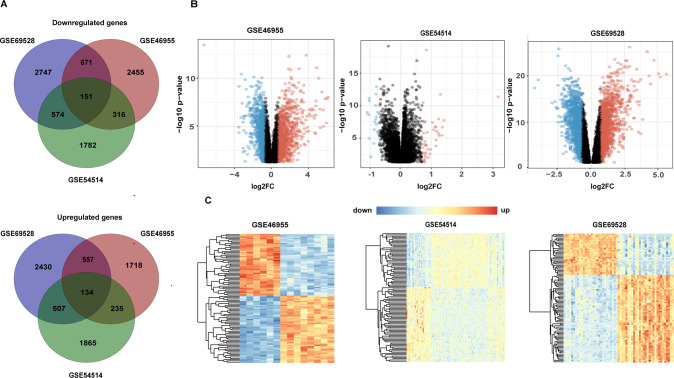
Differential expression analysis of the GSE69528, GSE46955, and GSE54514 datasets. **A** Venn diagram showing the overlapping DEGs in the three datasets. **B** Volcano plot shows significantly differentially expressed genes in the three datasets. Red and blue plots represent upregulated and downregulated genes, respectively, with (|log2FC | >0.7 and *P*-value<0.05). Black plots represent the genes with no significant difference. **C** Heatmap of the top 100 DEGs in three datasets. Each row represents a DEG, and each column represents a sample. The color ratio indicates the relative level of DEG expression: blue, lower than reference expression; red, higher than reference expression.

We performed differential gene expression analysis from sepsis patients in the GSE69528, GSE46955, and GSE54514 datasets. With a *P*-value set to <0.05 and | log2FC | of 0.7, we identified 7771, 6237, and 5564 DEGs from the GSE69528, GSE46955, and GSE54514 datasets, respectively (Supplementary Tables 4–6) and a set of DEGs overlapping in the three datasets (Fig. 2A). Volcano plots of up- and downregulated genes are shown in Fig. 2B, and the heatmaps of the top 100 DEGs in the three datasets are shown in Fig. 2C.

### Detection of sepsis pathway signatures

After searching sepsis-related literature in biomedical databases, 17 signaling pathways related to the pathogenesis of sepsis or important for the prognosis of sepsis patients were identified (Supplementary Table 1). These pathways may contain key pathogenic genes in sepsis, so we regarded them as feature pathways of sepsis. Disease signatures were constructed based on the DEGs derived from the three datasets and 17 signaling pathways (see gene IDs and logFC in Supplementary Tables 7–9). After integrating the logFC of DEGs in the three datasets with the genes in each pathway, calculating an average logFC for all genes due to pathway upregulation or downregulation and calculating the average logFC of the pathways corresponding to the three datasets (Supplementary Table 1), the TNF signaling pathway was identified as most upregulated in sepsis.

### Selection of potential sepsis drugs through the CTD benchmark

The CTD provides information on the relationship between genes, compounds, and diseases [13], and we used the CTD to query the association between compounds and sepsis. The drug lists related to the three datasets GSE46955, GSE69528, and GSE54514 were sorted, and the top 60 drugs were selected and verified in the CTD. Drugs were divided into four categories: those associated with sepsis, those not in the CTD, no disease data for the compound, and not associated with sepsis. If the drug was related to sepsis in the CTD, other studies have reported a relationship between the drug and sepsis pathogenesis. Since the purpose of this study was to find new drugs with therapeutic effects in sepsis, we regarded all other drugs except those related to sepsis in the CTD as new candidates for sepsis. Using this approach, the top 60 drugs related to the three datasets were identified (Supplementary Table 2).

**Fig. 3:**
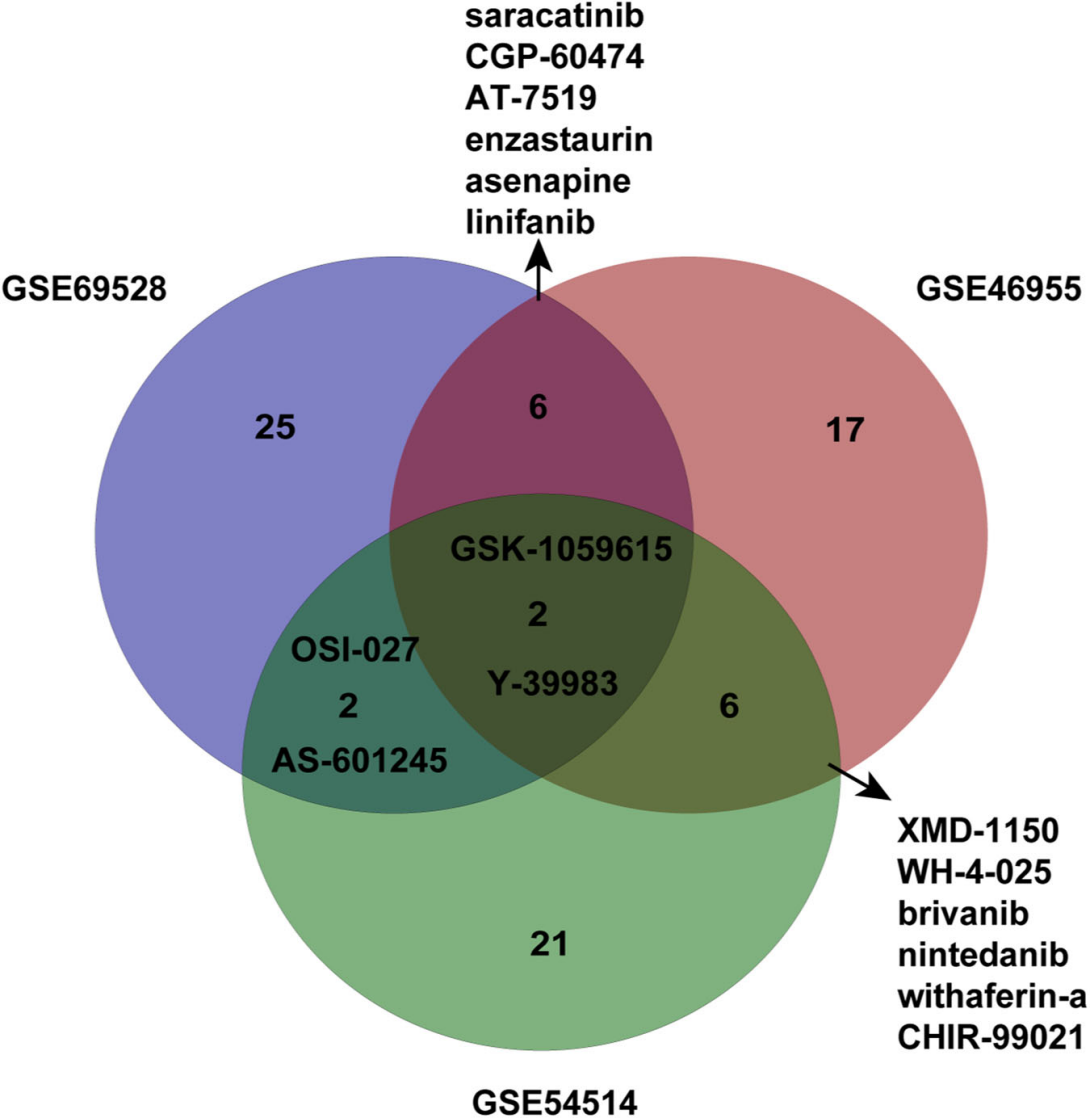
Overlap of three candidate drug lists. Venn diagram showing candidate drugs for sepsis obtained from the three datasets. Sixteen drugs appeared in at least two results.

Sixteen candidate drugs were included in two or three drug lists: Y-39983, CHIR-99021, WH-4-025, brivanib, XMD-1150, CGP-60474, saracatinib, enzastaurin, withaferin-a, AT-7519, linifanib, asenapine, nintedanib, AS-601245, GSK-1059615, and OSI-027. Three were previously reported to have anti-sepsis effects: CGP-60474 [14], AT-7519 [15], and Y-3998 [16], eleven drugs had not yet been reported, and no information was available for two drugs (WH-4-025, XMD-1150). Finally, we obtained the overlap of candidate drugs (Fig. 3).

### Experimental validation of potentially anti-sepsis drugs in vitro

**Fig. 4:**
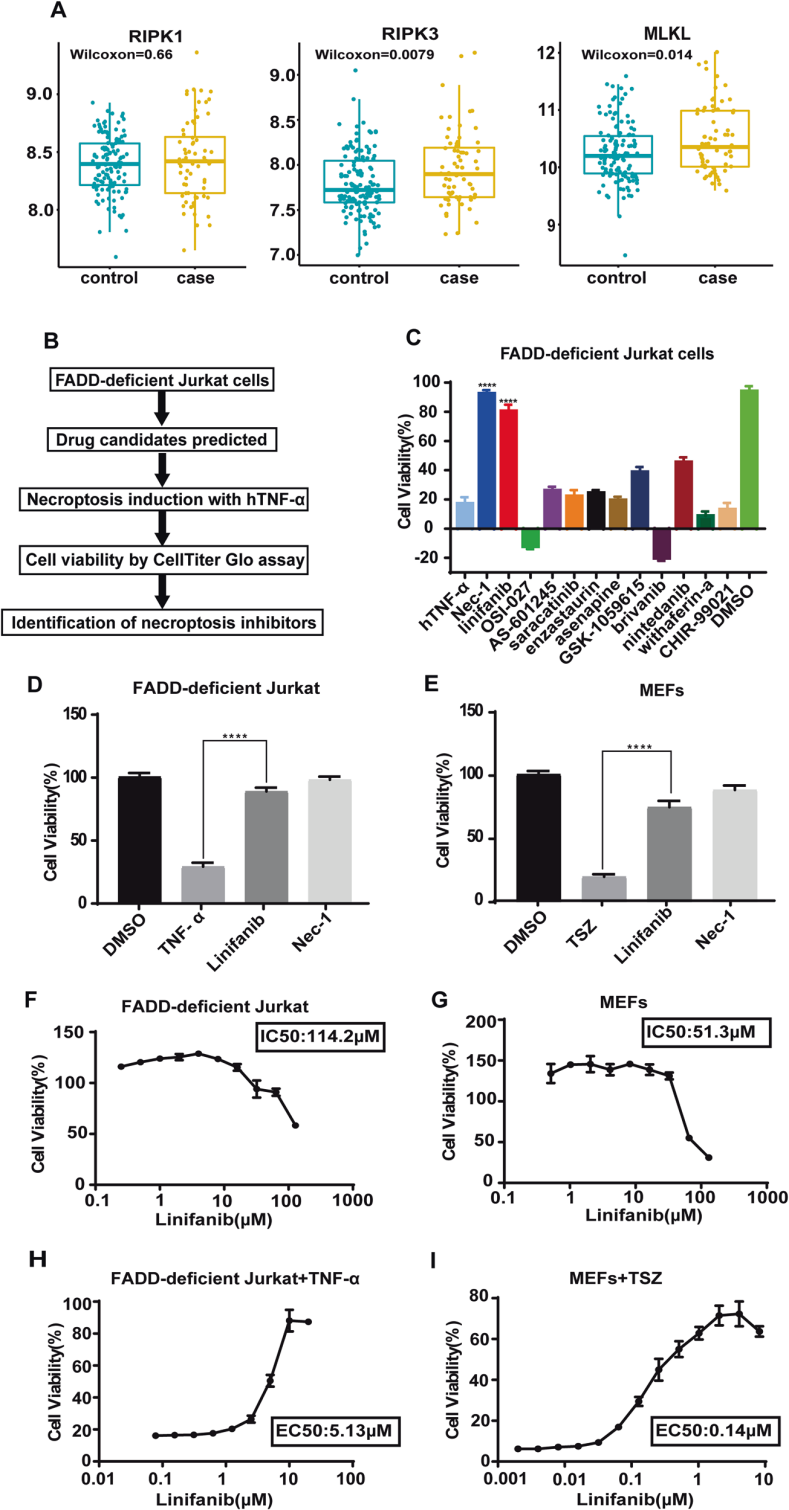
Validation of candidate drug by cellular assay identifies linifanib as a necroptotic inhibitor. **A** Boxplots for the focused genes (RIPK1, RIPK3 and MLKL) of necroptosis pathway in three sepsis datasets. **B** Schematic overview of the drug screening workflow. **C** Identification of necroptosis inhibitors using candidate drugs predicted with the LINCS and CTD databases for sepsis treatment based on bioinformatics. FADD-deficient Jurkat cells were pretreated with each compound (10 µM) for 30 min and then stimulated with human TNF-α (50 ng/ml) overnight to induce necroptosis. Cell viability was analyzed by ATP assay and normalized to untreated control cells (DMSO = 100). Nec-1 (25 µM) was used as a positive control. Cell viability was detected in FADD-deficient Jurkat cells (**D**) and MEF cells (**E**) that were pretreated with linifanib (10 µM) for 30 min in the presence or absence of 25 µM Nec-1 and then stimulated with 50 ng/ml human TNF-α or TSZ: TNF‐α (25 ng/ml) plus Smac mimetic (200 nM) and z‐VAD‐fmk (20 μM) for 24 h. Dose-dependent protection of linifanib (0.05 µM–100 µM) in (**F**) FADD-deficient Jurkat cells and (**G**) MEF cells. Cell viability was determined by dose-dependent protection of linifanib against TNF-α-induced necroptosis in (**H**)FADD-deficient Jurkat cells treated with various concentrations of linifanib (0.01 µM–20 µM) for 30 min followed by stimulation with human TNF-α (50 ng/ml) for 24 h, while (**I**) MEF cells were preincubated with linifanib (concentrations as indicated) for 30 min with 25 ng/ml mTNF-α (T) together with 200 nM Smac mimetic (S) and 20 μM caspase inhibitor z-VAD (Z) -fmk for 24 h. Cell viability was assessed using a luminescence-based readout for ATP (CellTiter Glo). Data represent mean value ±S.D. of two independent experiments and normalized to untreated control. ***p < 0.01, and ****p < 0.0001.

Necroptosis plays a significant role in the pathophysiology of SIRS and sepsis. Receptor-interacting serine/threonine-protein kinase 1 (RIPK1) regulates RIPK3-MLKL-driven systemic inflammation, and RIPK1 kinase inhibitors have shown promise in alleviating or preventing a SIRS response. We found significantly higher expression of RIPK1, RIPK3, and MLKL in the peripheral blood nucleated cells of sepsis patients than controls (Fig. 4A).

To further explore the potential novel therapeutic targets identified by our bioinformatics predictions, we performed phenotypic screening for inhibitors of TNF-α-induced necroptosis in FADD-deficient Jurkat cells. FADD-deficient Jurkat cells were pretreated with or without the top 11 candidate compounds (10 μM, 30 min) and then stimulated with 50 ng/ml TNF-α for 24 h or left untreated [17]. Nec-1 was used as a positive control to monitor RIPK1 kinase dependency in the induced necroptosis [18]. Cell viability was determined using the ATP-based CellTiter-Glo^®^ Luminescent Assay (Promega, Madison, WI). Measurements were normalized to untreated control cells (100%) (Fig. 4B). One of the top 11 candidate drugs (linifanib) effectively protected cells from necroptosis (Fig. 4C–E).

We next performed dose-response experiments to quantitatively assess the inhibitory potency of linifanib according to the half maximal inhibitory concentration (IC_50_), which was 114.2 µM in FADD-deficient Jurkat cells (Fig. 4F) and 51.3 µM in mouse embryonic fibroblasts (MEFs) (Fig. 4G). The half maximal effective concentration (EC_50_) for inhibiting necroptosis was 5.13 μM in human FADD-deficient Jurkat cells (Fig. 4H) and 0.14 µM in MEFs (Fig. 4I). Taken together, these data demonstrate that linifanib efficiently blocked TNF-α-induced necroptosis in both human and murine cells and is a potent necroptosis inhibitor.

### Linifanib blocks necrosome formation by inhibiting RIPK1 phosphorylation

**Fig. 5:**
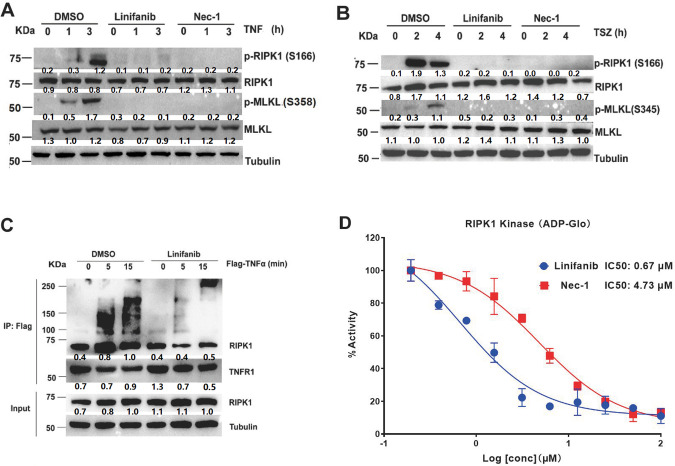
Linifanib prevents RIPK1-mediated cell death and is a RIPK1 kinase inhibitor. **A** FADD-deficient Jurkat cells were pretreated with linifanib (10 µM), Nec-1 (25 µM) or DMSO for 30 min followed by stimulation with hTNF-α (50 ng/mL) for the indicated time. The expression levels and activation status (phosphorylation) of the indicated necrosome members p-RIPK1^S166^ and p-MLKL^S345^ were analyzed by western blotting using the indicated specific antibodies. Data shown are representative of two independent experiments. **B** MEFs cells were pretreated with DMSO, linifanib (5 µM), Nec-1 (25 µM) plus Smac mimetic (200 nM) and z-VAD-fmk (20 µM) followed by stimulation with mTNF-α (25 ng/mL) for the indicated durations. **C** TNF-α-induced complex I immunoprecipitation (IP) of MEFs cells treated in the absence or presence of linifanib (0.31 µM) with Flag-mTNF-α (100 ng/ml) for the indicated time points, followed by anti-Flag beads and western blotting analysis using the indicated antibodies. Lysates pre-IP (input) were also analyzed by western blotting using the indicated antibodies. **D** In vitro ADP-Glo™ kinase assays using recombinant human RIPK1 protein (200 nM). Recombinant hRIPK1 was incubated with linifanib and Nec-1 at the indicated concentrations. Data represent mean value ±S.D. of two independent experiments.

The protective effect of linifanib against TNF-α-induced necroptosis was as potent as that of the well-established but experimental RIPK1 inhibitor Nec-1 [19]. We next evaluated whether linifanib inhibited necroptosis via RIPK1. FADD-deficient Jurkat cells were pretreated with 4 μM linifanib or 25 μM Nec-1 for 30 min followed by treatment with or without TNF-α for the indicated time points. Linifanib selectively inhibited RIPK1 and MLKL phosphorylation when necroptosis was induced by TNF-α in FADD-deficient Jurkat cells (Fig. 5A). Similar results were also obtained in murine MEFs (Fig. 5B). Thus, linifanib inhibits TNF-α-induced phosphorylation of RIPK1 and MLKL.

TNF-α can rapidly activate TNFR1 upon binding, thereby inducing recruitment of RIPK1 to TNFR1 to form a TNFR1 signaling complex (TNF-RSC, or complex I) [7, 20, 21]. The impact of linifanib on complex I formation was analyzed by immunoprecipitation (IP) after stimulation with the anti-TNFR1 antibody FLAG-tagged TNF-α in MEFs [19, 22]. Recruitment of RIPK1 into the TNF-RSC was largely blocked by linifanib in TNF-α-stimulated MEFs.

We next conducted an in vitro kinase assay to further test the potency of linifanib to directly inhibit RIPK1. Quantification of the ADP-Glo kinase assay showed that linifanib suppressed recombinant hRIPK1 [23] kinase activity (IC_50_ = 0.67 μM) (Fig. 5D). Our findings indicate that linifanib is as effective as the well-established RIPK1 inhibitor Nec-1 in preventing necroptosis and is a RIPK1 kinase inhibitor.

### Linifanib protects mice from TNF-α-induced SIRS

**Fig. 6:**
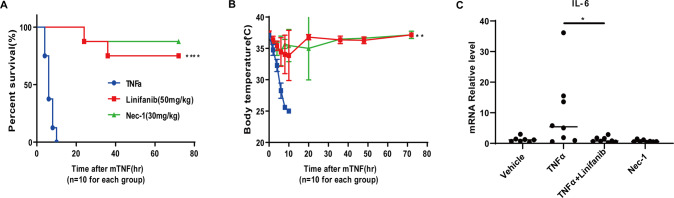
Linifanib protects mice against SIRS induced by TNF-α. Ten-week-old male C57BL/6 J mice were pretreated with or without linifanib (50 mg·kg-1 gavage) or Nec-1 (30 mg·kg-1 gavage) for 30 min, and then SIRS was induced with mTNFα (5 μg/ mice i.v.). Control mice received an equal amount of vehicle before mTNF-α challenge. **A** The survival curve and **B** the mouse body temperatures (means ± SEM) of linifanib-treated mice (*n* = 10 for each group), Nec-1-treated mice (*n* = 10 for each group), and vehicle control mice (*n* = 10 per group) are shown. The results are presented as the mean value ±S.D. **p < 0.01, ****p < 0.0001. **C** After SIRS induction for 6 h, the IL-6 levels of lung tissues were determined by real-time PCR. Results represent the mean±S.D. from six mice. **p < 0.01, significantly different from vehicle control group.

To explore whether linifanib protects against inflammation in vivo, we treated TNF‐induced SIRS mice with the drug [6]. Linifanib (50 mg/kg) or Nec-1 (30 mg/kg) given by intragastric gavage 30 min before i.v. injection of mTNF‐α protected mice from hypothermia and death (Fig. 6A, B). Since interleukin 6 (IL-6) levels in vivo correlate well with serum TNF‐α levels and the mortality rate of patients with septic shock [24–26], we quantified IL-6 in the lungs of SIRS mice. IL-6 expression significantly increased in the lung tissue 6 h after receiving TNF‐α injection, and IL-6 overexpression in SIRS mice was significantly suppressed by pretreatment with linifanib (Fig. 6C). Thus, linifanib protects against TNF‐induced SIRS in vivo.

## Discussion

Sepsis is a life-threatening illness occurring due to a severe and systemic inflammatory response to infection [27]. The syndrome is initially characterized by dysregulated inflammation, namely SIRS, which can rapidly progress to severe sepsis and septic shock [28]. Sepsis is the most common cause of death in critically ill patients in noncoronary intensive care units [29]. Necroptosis, a potent proinflammatory mechanism triggered by TNF-α in response to tissue injury and inflammation [30], plays a vital role in the pathophysiology of systemic sepsis [6, 31]. Indeed, most information regarding necroptosis has been derived from studies of TNF-α-induced necroptosis [32–34]. Using bioinformatics analysis and a drug repositioning strategy, we predicted a set of potential drug candidates for sepsis and validated the effectiveness of one of these drugs in vitro and in vivo models of TNF-α-induced necroptosis. Our experimental validation found that the candidate drug linifanib effectively inhibited necroptosis, SIRS, and related tissue damage.

We developed a workflow to optimize our drug repositioning strategy. First, we predicted therapeutic drugs by analyzing existing gene expression datasets of patients with sepsis, which was an efficient and cost-effective approach compared with traditional experiments. Second, by analyzing the pathophysiological mechanisms underpinning sepsis, we established an effective experimental verification system for the predicted drug candidates. Third, we used the LINCS database containing an FDA-approved drug library, and in doing so repurposed linifanib to inhibit necroptosis, related inflammation, and SIRS. Since the safety evaluation of linifanib has already been completed, clinical translation should be expedited.

Sepsis-induced acute lung injury remains the main cause of death in septic patients [35]. Necroptosis is activated in the lung tissue of septic patients and is also caused by SARS-CoV-2 [36]. RIPK1 kinase plays a crucial role in mediating necroptosis upon activation of TNFR1 by TNF-α [32]. Upon necrosome formation, RIPK1 is activated and autophosphorylated, activating RIPK3, which binds to and phosphorylates the pseudokinase MLKL, mediating necroptosis in disease states [37, 38]. We found that the expression of RIPK1, RIPK3, and MLKL was much higher in the peripheral blood nucleated cells of septic patients than controls. This provides further evidence that necroptosis plays a significant role in the pathophysiology of sepsis and suggests that RIPK1-dependent necroptosis may be a potential novel therapy target in sepsis.

Our data illustrate that Linifanib increases recruitment to complex I and inhibits RIPK1. To validate the capacity of linifanib to inhibit RIPK1, we used the ADP-Glo™ kinase assay to evaluate the IC50 of linifanib. The ADP-Glo™ Kinase assay measures kinase activity by quantifying ADP production during the kinase reaction. The assay is well suited for measuring the effects of compounds on the activity of many purified kinases. We found that linifanib directly inhibited RIPK1 kinase activity in a dose-dependent manner with an IC50 of 0.67 μM, seven-fold lower than that of the necroptosis inhibitor Nec-1 as a positive control, so linifanib either suppresses RIPK1 activation directly or indirectly with excellent prospects for clinical application.

Necroptosis is characterized by marked cellular swelling, plasma membrane rupture, and subsequent damage-associated molecular pattern (DAMP) released after cellular disruption [39]. TNF-α-induced necroptosis initiates downstream signaling cascades driving the production of a series of proinflammatory cytokines, including IL-6 [40]. Elevated concentrations of IL-6 trigger a detrimental cytokine storm [41] in sepsis and are negatively associated with poor clinical outcomes [42]. Endothelial cell necroptosis may contribute to lethality both in SIRS and septic patients [43]. Therefore, we used a murine SIRS model induced by a tail vein injection of TNF-α to confirm that linifanib can effectively rescue shock-related death and inhibit overexpression of IL-6 in the damaged lung tissue of SIRS mice. These data provide a solid basis for the clinical application of linifanib in the treatment of sepsis.

This study has limitations. First, despite recent research progress, our understanding of SIRS pathogenesis is still incomplete. More studies on the pathophysiological mechanisms of sepsis and suitable validation systems for therapeutic targets of sepsis are needed. Second, whether linifanib can inhibit sepsis-related inflammatory responses and reduce mortality in patients still needs to be proven in clinical trials. Linifanib is still an expensive, patent-protected drug, limiting its current application to investigator-initiated clinical trials and translational research. Lastly, since the drug prediction is based on sepsis, linifanib requires further validation in other authentic sepsis models such as LPS treatment or cecal ligation and puncture in vivo.

In conclusion, linifanib inhibited RIPK1-dependent necroptosis and attenuated SIRS-induced sepsis, providing the first preclinical data supporting repurposing linifanib to reduce mortality from sepsis.

## Experimental verification

### Biological reagents

We used the following reagents: Flag-mTNF-α (ME15JA468, Sino Biological, Beijing, China) was a gift from Professor Daichao Xu (Interdisciplinary Research Center on Biology and Chemistry, Shanghai); recombinant murine/human TNF-α(Sino Biological, 50349-MNAE) and (Peprotech, 300-01A-100); Linifanib (Selleck Chemicals, S1003, CAS# 796967-16-3); Pierce protease inhibitor tablets (Thermo Fisher Scientific, A32965); z-VAD-fmk (Selleck Chemicals, S7023); Smac mimetic (SM-164) (Beyotime Biotechnology, SC0114-10 mM); Necrostatin-1 (Selleck Chemicals, S8037); CellTiter-Glo Luminescent Cell Viability Assay (Promega, G7573); FDA-approved chemical library (Selleck Chemicals, L1300); ANTI-FLAG® M2 Affinity Gel (Sigma, A2220); ADP-Glo Kinase Assay (V6930, Promega, Madison, WI); RIPK1 Kinase Enzyme System (VA7591, Promega); Protein A/G ultra link resin (Thermo Fisher Scientific, 53133); Pierce Protease Inhibitor Tablets (Thermo Fisher Scientific, A32965). The antibodies used for western blotting were obtained from commercial sources: anti-RIPK1 (BD Biosciences, 610459 and Cell Signaling Technology, 3493 S); anti-mouse phosphoS166-RIPK1 (Cell Signaling Technology, Cat# 31122 S); anti-mouse MLKL (Proteintech, 66675-1-Ig); anti-mouse-phosphoS345-MLKL (Abcam, ab196436), anti-Tubulin (Proteintech, 66031-1-Ig); anti-TNFR1 (Proteintech, 21574-1-AP); anti-human phospho-RIPK1 S166 (Cell Signaling Technology, Cat# 65746), anti-human MLKL (Abcam, Cat# ab183770), and anti-human phospho-MLKL (Abcam, Cat# ab187091).

### Cells and cell culture

Mouse embryonic fibroblasts (MEFs) and FADD-deficient Jurkat cells were kindly provided by Prof. Daichao Xu of Interdisciplinary Research Center on Biology and Chemistry, CAS, Shanghai, China. FADD-deficient Jurkat cells were cultured in RPMI 1640 medium (Hyclone), MEFs were cultured in DMEM (Hyclone). The medium was additionally supplemented with 10% FBS (Zhejiang Tianhang Biotechnology), MEM (HyClone), NEAA (HyClone), and antibiotics (100 U/ml penicillin and 100 mg/ml streptomycin) (Gibco). All cell lines were cultured in a humidified 5% CO2 atmosphere at 37 °C.

### Necroptosis induction and cell viability analysis

For FADD-deficient Jurkat cells, necroptosis was induced by human TNF-α (50 ng/ml) for 24 h. For MEF cells, necroptosis was induced by pretreatment with Smac164 (200 nM) and Z‐VAD‐fmk (20 μM) for 30 min, followed by mTNF‐α (25 ng/ml). The compounds were incubated with the cells at the indicated concentrations for 24 h. Cell viability was then examined by using the CellTiter‐Glo Luminescent ATP Assay kit (Promega, G7573). Luminescence or absorbance was recorded with a BioTek 312e microplate reader (BioTek Instruments, Winooski, VT).

### Animal experiments

All animal care and experimental procedures complied with the National Institutes of Health guidelines (NIH publications Nos. 80–23, revised 1996) and under the approval of the Ethical Committee of the China Three Gorges University Laboratory Animal Center. For the TNF‐induced SIRS model, male C57BL/6 J mice (8–10 weeks old) were purchased from Beijing Vital River Laboratory Animal Technology Co., Ltd. (Beijing, China). Mice were raised in an SPF facility with a pathogen‐free environment (23 ± 2 °C and 55 ± 5% humidity) with a 12:12 h light/dark cycle at the Three Gorges University Laboratory Animal Center. Mice were administered Nec-1/linifanib or solvent CMC-Na by p.o. gavage at the indicated doses 30 min before i.v. injection of TNF-α (5 μg). TNF-α was diluted in PBS and injected i.v. (5 µg/mouse) in a volume of 200 μl. Body temperature was monitored with an electric thermometer. Lung tissues were collected at the indicated times after sacrificing the mice.

### RNA extraction, reverse transcription, and real-time PCR

The mRNA levels of lung tissues from SIRS model mice were analyzed at 6 h after injection of TNF-α (5 μg/mouse). Total lung RNA was extracted with the FastPure Cell/Tissue Total RNA Isolation Kit V2 (RC112-01, Vazyme) and reverse transcribed into cDNA using HiScript III RT SuperMix (R323-01, Vazyme). Quantitative real-time PCR was performed using ChamQ SYBR qPCR Master Mix (Q311-02, Vazyme) and analyzed with CFX Manager software from Bio–Rad CFX384. Relative gene expression was normalized to 18 s rRNA and determined using the -ΔΔCT method. Primers used for 18 s, forward:5′-AGTCCCTGCCCTTTGTACACA-3′, reverse: 5′-CGATCCGAGGGCCTCACTA-3′; for IL-6, forward: 5′-TACCACTCCCAACAGACCTG-3′, and reverse:5′-GGTACTCCAGAAGACCAGAGG - 3′.

### Western blotting analysis

Cells were lysed in 1% NP-40 buffer for total lysis (1% Nonidet P-40, 50 mM Tris Base (pH 7.5), 150 mM NaCl, 1 mM PMSF, 1 mM NaF/Na3VO4, Pierce Protease Inhibitor Tablets). All cell lysis buffers were supplemented with 1% NP-40 buffer to the same concentration and were then added to 5x loading buffer (10% SDS, 40% glycerol, 25% 1 M Tris-HCl (pH 6.8), 0.005% bromophenol blue, 25% β-mercaptoethanol) with denaturation at 95 °C for 5 min. Cell lysates were separated on SDS‐PAGE gels with running buffer and subsequently transferred onto immobilon-NC transfer membranes (NC membrane, HATF00010, Millipore) or 0.45 μM polyvinylidene difluoride membranes (PVDF membrane, MXHVWP124, Millipore). Membranes were blocked in 5% skimmed milk in Tris-buffered saline (TBS) containing 0.1% Tween 20 before overnight incubation with specific primary antibodies at 4 °C. All listed primary antibodies were used at 1:1000 dilution. The membranes were then washed and incubated with the appropriate HRP-conjugated secondary antibodies (1031-05 and 4050-05, SouthernBiotech), developed immunoreactivity (G2014-50ML, Servicebio), and imaged using a Tanon-4800 (Tanon Science & Technology Co., Ltd.).

### Immunoprecipitation

For immunoprecipitation (IP) of complex I, MEFs were lysed with 1% NP-40 buffer (1% Nonidet P-40, 50 mM Tris Base (pH 7.5), 150 mM NaCl, 1 mM PMSF, 1 mM NaF/Na3VO4 and Pierce Protease Inhibitor). Complex I was purified by IP using Anti-Flag® M2 Affinity Gel (20 µl). The IP protein was rotated overnight at 4 °C. The beads were washed three times with ice-cold 1% NP-40 buffer and the IP protein rotated overnight at 4 °C. After incubation with cell lysates, beads were washed with ice-cold 1% NP-40 buffer and eluted by directly adding 2x loading buffer (4% SDS, 20% glycerol, 10% 1 M Tris-HCl (pH 6.8), 0.005% bromophenol blue, 10% β-mercaptoethanol). Samples were incubated at 95 °C for 5 min and analyzed by immunoblotting (antibodies used as indicated).

### In vitro kinase assays

We followed the protocol of the RIPK1 Kinase Enzyme System (Promega, Cat# VA7591) and ADP-Glo™ Kinase Assay (Promega, Cat# V6930) to detect the inhibition of RIPK1 kinase activity by linifanib.

### Data and statistical analysis

Results are presented as means ± SEM. Data were analyzed using GraphPad Prism 8 (GraphPad Software, La Jolla, CA, USA). Statistical analyses used were the t-test or 1- or 2-way ANOVA for two or more than two groups, respectively. The log-rank test was performed for Kaplan-Meier survival curve analysis.

## Materials and methods

### Drug prediction

#### Data collection

Datasets associated with sepsis were retrieved and downloaded from the Gene Expression Omnibus (GEO, http://www.ncbi.nlm.nih.gov/geo/) database using a keyword of “sepsis”, organism “*Homo sapiens*”, and study type “expression profiling by array”. The GSE69528 [44], GSE46955 [45], and GSE54514 [46] datasets were retrieved (Supplementary Table 3) and used to analyze differentially expressed genes.

#### Identification of differentially expressed genes (DEGs)

DEG analysis was conducted using *limma* (v3.46.0) in R (version x64 4.0.3). Gene expression data were log_2_ transformed and processed in R. To ensure sufficient overlapping genes after intersecting DEGs and signaling pathway genes to construct disease signatures, we used a *P*-value <0.05 to screen DEGs. The heatmaps of the top 100 DEGs were produced using the *pheatmap* package (v1.0.12) in R.

#### Identification of signaling pathways

To identify the key pathogenic signaling pathways associated with sepsis, we searched the PubMed database using the keyword “sepsis” and identified signaling pathways closely related to the pathogenesis of sepsis from the literature.

#### Perturbation response signatures

The Library of Integrated Network-Based Cellular Signatures (LINCS) is a library cataloging the functional genomics and drug metabolomics of human cell lines treated with small molecules and quantification of multilevel cell responses before and after treatment [47]. The LINCS L1000 library exploits correlations between gene expression; using small molecules to treat human cell lines, they identified 978 genes as genome-wide markers in 384-well plates based on large-scale statistical analysis. The expression of other genes can then be calculated by measuring the expression of marker genes. Changes in the nearly 1000 genes adopted by L1000 represent approximately 80% of gene change information in humans [48].

Drug perturbation genesets in LINCS can be divided into three groups: (1) landmark space, containing 978 landmark genes based on probe fluorescence intensity data measured experimentally, scaled, and standardized after calculation with the control group; (2) all inferred genes, containing 978 landmark genes and 11,350 genes whose expression data are inferred, totaling 12,328 genes; and (3) best inferred genes, which contain 978 landmark genes and 9196 high-fidelity inferred genes from 11,350 inferred genes, totaling 10174 genes used in drug prediction.

To identify potential therapeutic drugs for sepsis, we obtained the gene expression profiles perturbed by small molecule compounds from the LINCS L1000 level 5 dataset. The perturbation data of each small molecule compound included the gene expression data of different cell lines after different doses and treatment times.

#### Kolmogorov–Smirnov test

The Kolmogorov–Smirnov test (KS test) is a nonparametric test based on a cumulative distribution function to test whether an empirical distribution conforms to a theoretical distribution or to compare whether there is a significant difference between two empirical distributions [49, 50].

*X*_*1*_*, X*_*2*_*,…, X*_*m*_, *Y*_*1*_*, Y*_*2*_*,…, Y*_*n*_ are two independent random samples, and the distribution functions are *F*_*m*_(*t*) and *G*_*n*_(*t*), which can be defined as:

1 $$\begin{array}{*{20}{c}} {F_m\left( t \right) = \frac{1}{m}\mathop {\sum }\limits_{i = 1}^m I_{\left[ { - \infty ,t} \right]}\left( {X_i} \right)} \end{array},$$

2 $$\begin{array}{*{20}{c}} {G_n\left( t \right) = \frac{1}{n}\mathop {\sum }\limits_{i = 1}^n I_{\left[ { - \infty ,t} \right]}\left( {Y_i} \right),} \end{array}$$

where m and n denote the number of samples in the two groups, t denotes any real number, and $I_{\left[ { - \infty ,t} \right]}(X_i)$ and $I_{\left[ { - \infty ,t} \right]}(Y_i)$ are defined as:

3 $$\begin{array}{l} {I_{\left[ { - \infty ,t} \right]}\left( {X_i} \right) = \left\{ {\begin{array}{l} {1,X_i \,\le\, t} \\ {0,X_i \,>\, t} \end{array}} \right.,} \end{array}$$

4 $$\begin{array}{l} {I_{\left[ { - \infty ,t} \right]}\left( {Y_i} \right) = \left\{ {\begin{array}{l} {1,Y_i \,\le\, t} \\ {0,Y_i \,>\, t} \end{array}} \right..} \end{array}$$

The KS test is defined as:

5 $$\begin{array}{*{20}{c}} {D_{m,n} = sup_t\left| {F_m\left( t \right) - G_n\left( t \right)} \right|,} \end{array}$$

where *sup*_*t*_ is the supremum of distance.

The KS test quantifies the distance between the empirical distribution functions of two groups of samples. The null hypothesis H_0_ states that the two data distributions are consistent or the data conform to the theoretical distribution. When the actual observed value *D*_*m,n*_ is greater than the critical value, then H_0_ is rejected; otherwise, H_0_ is accepted.

#### Calculation of therapeutic scores

We used DEGs in sepsis-related pathways to construct disease signatures and DEGs from drug action to construct drug signatures. The differential expression values of genes in sepsis-related pathways were from log-fold changes (logFC) after differential expression analysis of sepsis-related datasets, and the differential expression of genes under the action of drugs came from the L1000 library. Based on the KS test statistics, these disease and drug signatures were used to design a pattern matching method to calculate treatment scores for different drugs for sepsis.

For sepsis-related signaling pathway C and drug-induced gene expression profile D, the treatment scores of the upregulated geneset and downregulated geneset of pathway C relative to drug-induced gene expression profile D were calculated.

First, we took the intersection of genes upregulated or downregulated in sepsis-related pathways and genes changing after drug perturbation and recorded this gene set as H. Genes induced by drugs were arranged according to their differential expression from large to small, and genes in H were arranged from large to small according to this differential expression value. Using the KS test statistic, a and b are defined as:

6 $$\begin{array}{*{20}{c}} {a = \begin{array}{*{20}{c}} N \\ {\mathrm{max}} \\ {i = 1} \end{array}\left( {\frac{P}{N} - \frac{Q}{M}} \right),} \end{array}$$

7 $$\begin{array}{*{20}{c}} {b = \begin{array}{*{20}{c}} N \\ {\mathrm{max}} \\ {i = 1} \end{array}\left( {\frac{Q}{M} - \frac{{P - 1}}{N}} \right),} \end{array}$$

where N is the total number of genes in the H gene set, M is the total number of genes whose expression value changes after drug perturbation, P is the position of current gene g in gene set H, and Q is the position of gene g in the gene list under drug perturbation.

Therefore, the treatment score of the upregulated or downregulated geneset in pathway C is calculated as

8 $$\begin{array}{l} {Score_{up/down} = \left\{ {\begin{array}{l} {a,a > b} \\ { - b,a \le b} \end{array}} \right..} \end{array}$$

The treatment score of the drug for sepsis-related signaling pathway C is calculated as

9 $$\begin{array}{*{20}{c}} {Score^{c,d} = \left\{ {\begin{array}{*{20}{c}} {Score_{up}^{c,d} - Score_{down}^{c,d},Score_{up}^{c,d} \ast Score_{down}^{c,d} < 0} \\ {0,Score_{up}^{c,d} \ast Score_{down}^{c,d} \ge 0} \end{array}} \right.,} \end{array}$$

where c denotes the sepsis-related signaling pathway and d denotes the drug.

There are four possible effects of drugs on a sepsis-related signaling pathway. In the first case, the change in gene expression after drug perturbation is opposite to that in sepsis, so this drug has the potential to treat sepsis; in this case, *Score*^*c,d*^<0. In the second case, genes whose expression decreases in sepsis but increase or decreases under drug perturbation, the use of the drug in sepsis will return the expression of some genes to normal but others will decrease. In the third case, genes with increased expression in sepsis but the expression of some genes increases and others decreases after drug perturbation, so use of the drug in sepsis will cause the expression of some genes to return to normal but the expression of other genes will continue to increase. In the fourth case, the expression of genes increases or decreases in sepsis and in response to the drug, indicating that the effect of the drug and sepsis on gene expression changes is consistent and the drug may aggravate the severity of sepsis; in this case, *Score*^*c,d*^>0.

In the first and fourth cases, $Score_{up}^{c,d} \ast Score_{down}^{c,d} < 0$. After screening drugs that met these two conditions, drugs meeting the first condition were further screened according to *Score*^*c,d*^<0. The greater the absolute value of *Score*^*c,d*^, the more obvious the drug effect.

After calculating the drug score for each pathway, treatment scores were obtained by treating different cell lines with a drug, calculating the mean value of the score, and using its absolute value as the treatment score for the drug.

The average score of each drug in all pathways was calculated for each sepsis-related dataset. Only results with a score <0 were retained and sorted according to the absolute value of the score from large to small, including only drugs with potential therapeutic effects on sepsis. Finally, top 60 drugs overlapping in the three datasets were used to determine the final candidate drugs.

## Supplementary information

Supplymentary Table1

Supplymentary Table2

Supplymentary Table3

Supplymentary Table4

Supplymentary Table5

Supplymentary Table6

Supplymentary Table7

Supplymentary Table8

Supplymentary Table9

Original Data File

Ethics Statement

## Data Availability

The authors declare that all data generated and analysed during the current study are available at https://github.com/ykykyky/KS_Rcode.
