# Supplementary material for: Repositioning linifanib as a potent anti-necroptosis agent for sepsis
Source: Cell Death Discov. 2023 Feb 10;9:57. doi: 10.1038/s41420-023-01351-y (PMC9913023; doi:10.1038/s41420-023-01351-y)
Supplement: Supplementary file 1 — Supplymentary Table1 [file 41420_2023_1351_MOESM1_ESM.docx]

**Table 1. The mean value of logFC of DEGs in signaling pathways related to sepsis.**

| Pathway | Mean value of logFC |
| --- | --- |
| TNF signaling pathway | 0.513 |
| Cytokine-cytokine receptor interaction | 0.409 |
| NOD-like receptor signaling pathway | 0.377 |
| Toll Like Receptor 4 (TLR4) Cascade | 0.319 |
| NF-kappa B signaling pathway | 0.318 |
| JAK-STAT signaling pathway | 0.279 |
| C-type lectin receptor(CLR) signaling pathway | 0.270 |
| Necroptosis | 0.213 |
| PD-L1 expression and PD-1 checkpoint pathway in cancer | 0.160 |
| The NLRP3 inflammasome | 0.134 |
| PI3K-Akt signaling pathway | 0.104 |
| AGE-RAGE signaling pathway in diabetic complications | 0.103 |
| MAPK signaling pathway | 0.093 |
| STING mediated induction of host immune responses | 0.063 |
| T cell receptor signaling pathway | 0.017 |
| mTOR signaling pathway | 0.003 |
| PPAR signaling pathway | -0.069 |
