# Supplementary material for: Repositioning linifanib as a potent anti-necroptosis agent for sepsis
Source: Cell Death Discov. 2023 Feb 10;9:57. doi: 10.1038/s41420-023-01351-y (PMC9913023; doi:10.1038/s41420-023-01351-y)
Supplement: Supplementary file 2 — Supplymentary Table2 [file 41420_2023_1351_MOESM2_ESM.docx]

**Table 2. Distribution of the top 60 drugs of three datasets.**

|  | Associated with sepsis | No compounds in CTD | No disease data with compounds | Not associated with sepsis |
| --- | --- | --- | --- | --- |
| GSE46955 | 29 | 6 | 15 | 10 |
| GSE69528 | 26 | 10 | 8 | 16 |
| GSE54514 | 29 | 13 | 10 | 8 |
