# Supplementary material for: Repositioning linifanib as a potent anti-necroptosis agent for sepsis
Source: Cell Death Discov. 2023 Feb 10;9:57. doi: 10.1038/s41420-023-01351-y (PMC9913023; doi:10.1038/s41420-023-01351-y)
Supplement: Supplementary file 3 — Supplymentary Table3 [file 41420_2023_1351_MOESM3_ESM.docx]

**Table 3. Description of GEO datasets used in this study.**

| GEO accession | Case | Control | Species | Experimental Design |
| --- | --- | --- | --- | --- |
| GSE69528 | 29 | 28 | Homo sapiens | A Blood Transcriptional Diagnostic Assay for Septicemic Melioidosis |
| GSE46955 | 8 | 6 | Homo sapiens | Transcriptome analysis of blood monocytes from sepsis patients |
| GSE54514 | 31 | 96 | Homo sapiens | Whole blood transcriptome of survivors and nonsurvivors of sepsis |
