# Supplementary material for: Repositioning linifanib as a potent anti-necroptosis agent for sepsis
Source: Cell Death Discov. 2023 Feb 10;9:57. doi: 10.1038/s41420-023-01351-y (PMC9913023; doi:10.1038/s41420-023-01351-y)
Supplement: Supplementary file 10 — Original Data File [file 41420_2023_1351_MOESM10_ESM.pdf]

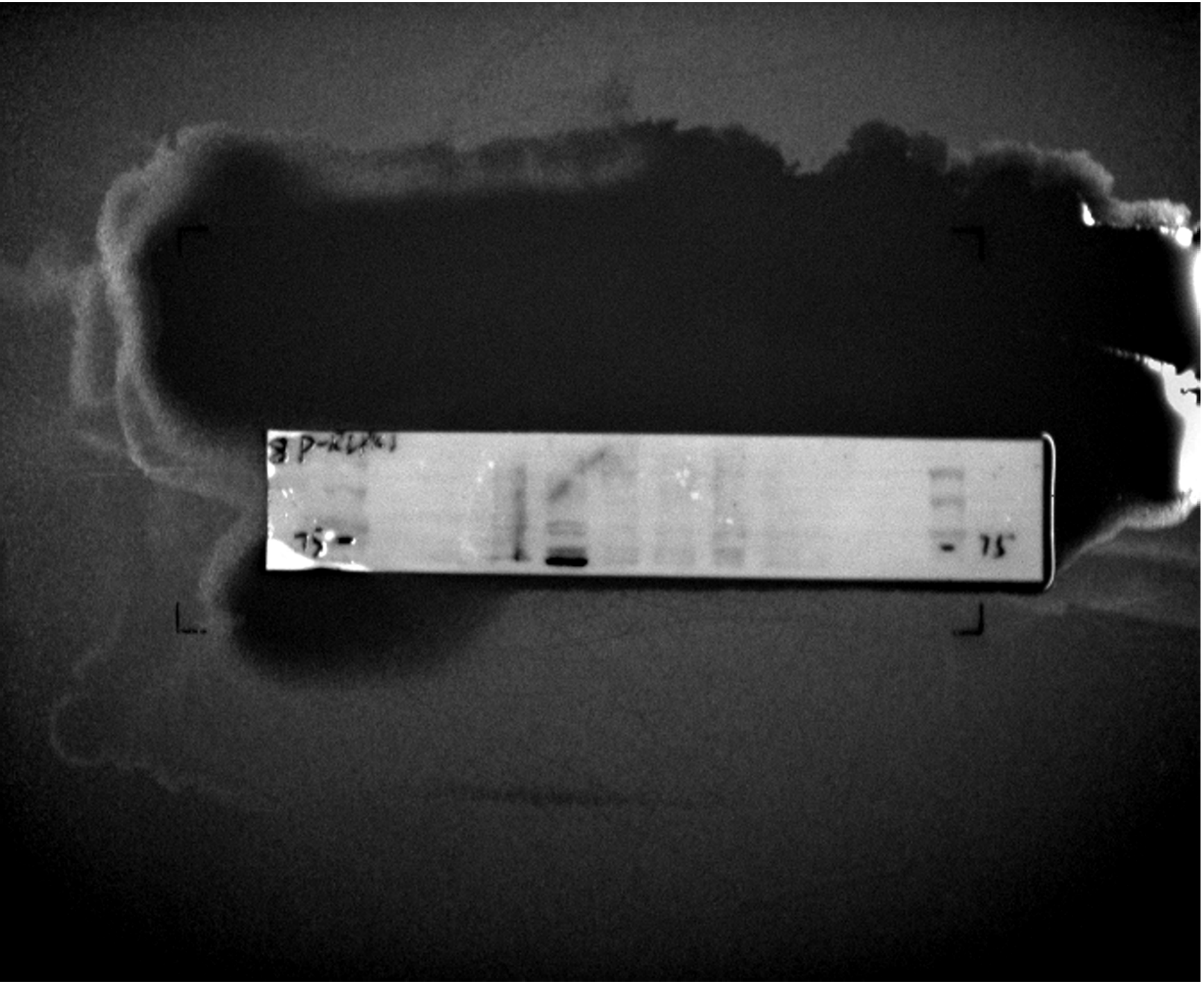

Figure5A FADD-deficient Jurkat-1 p-RIPK1(S166)

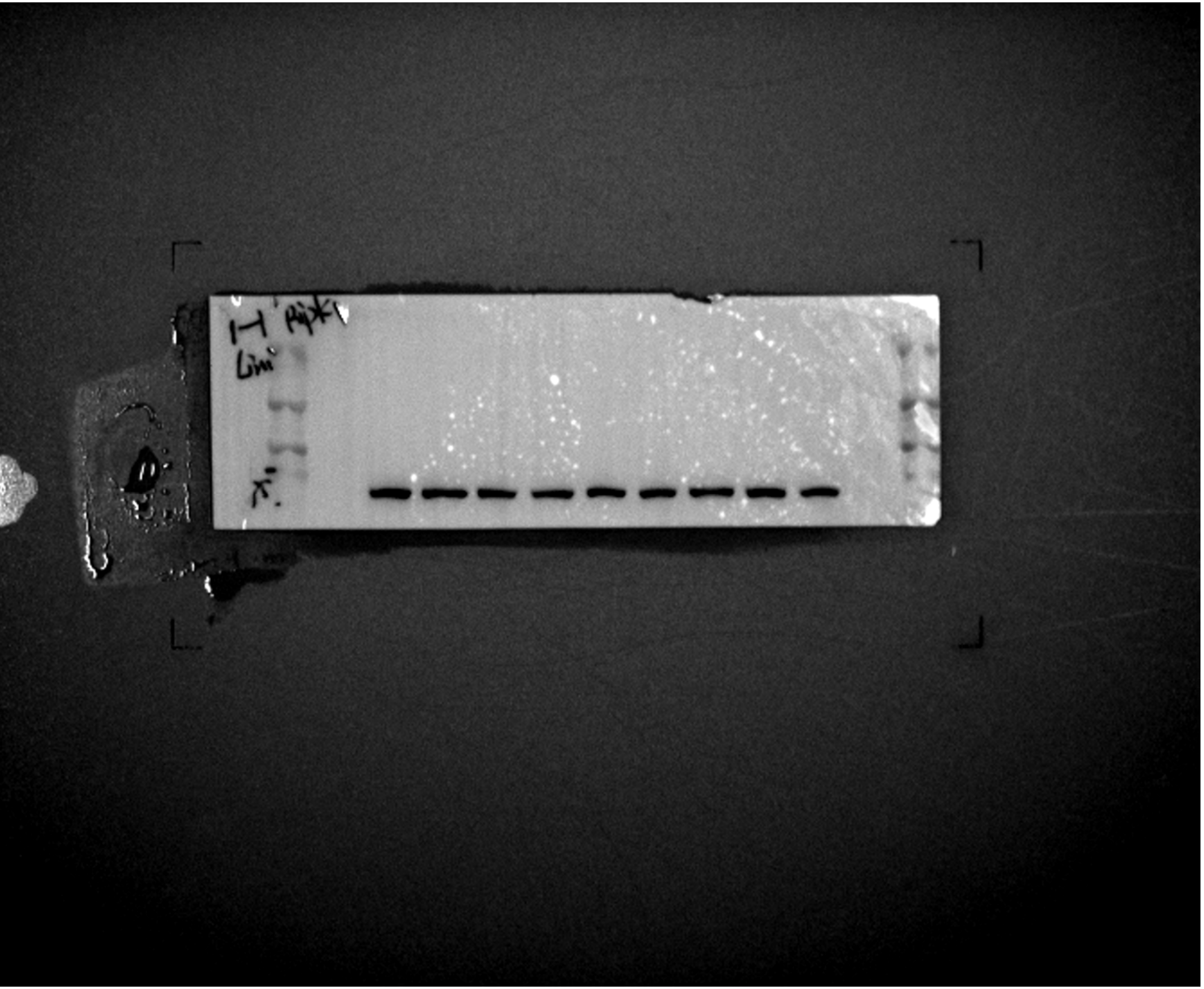

Figure5A FADD-deficient Jurkat-2 RIPK1

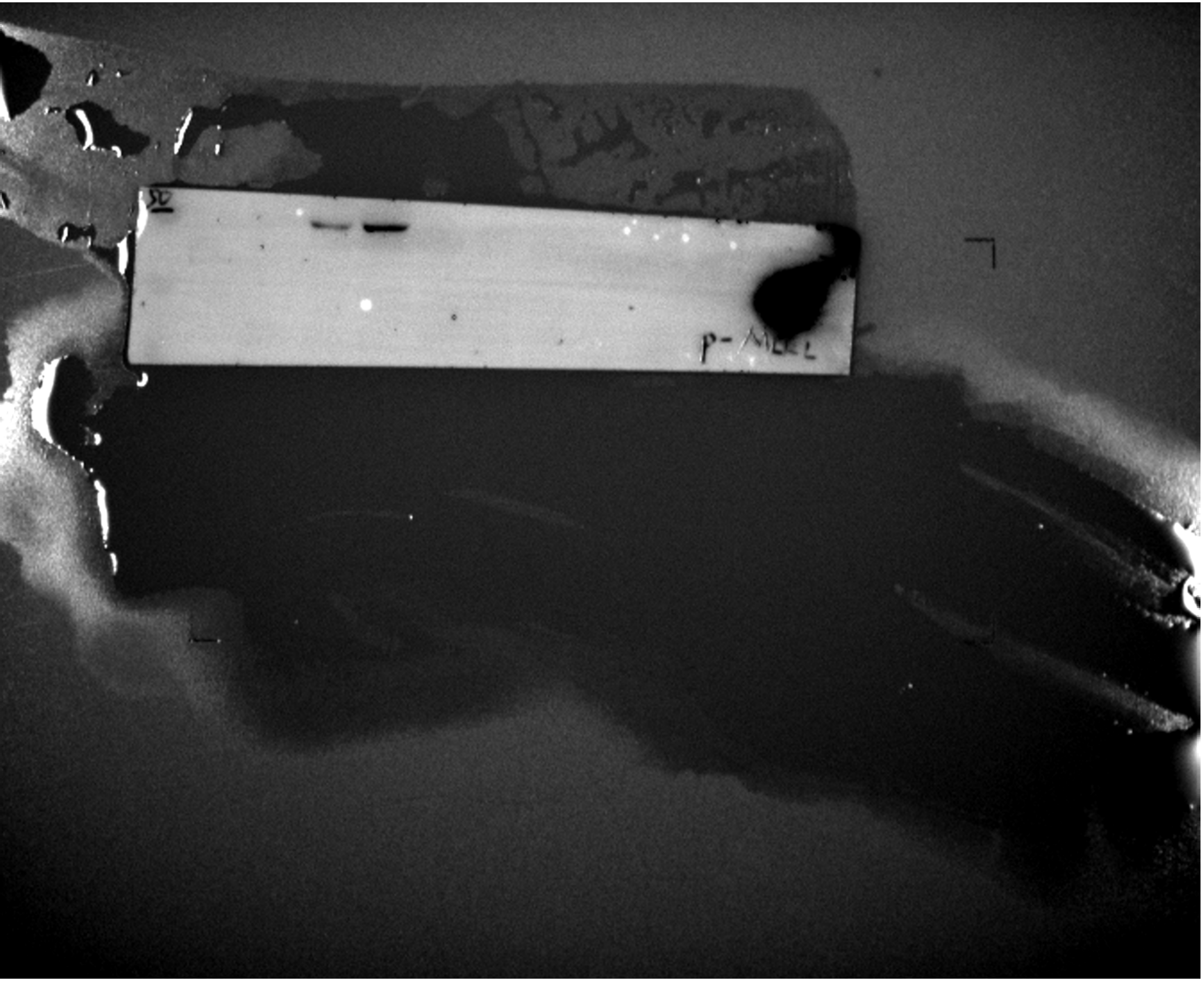

Figure5A FADD-deficient Jurkat-3 p-MLKL(S358)

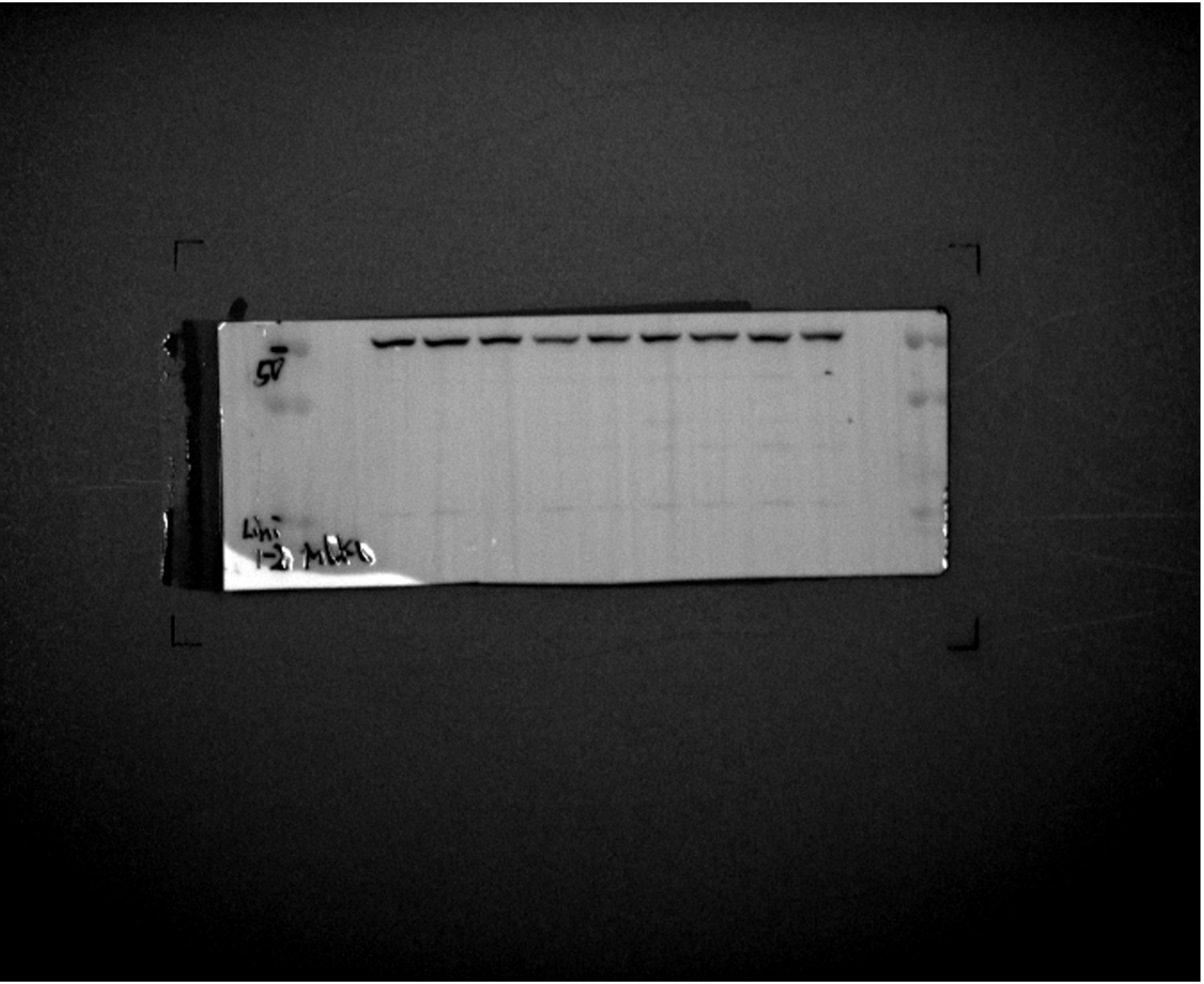

Figure5A FADD-deficient Jurkat-4 MLKL

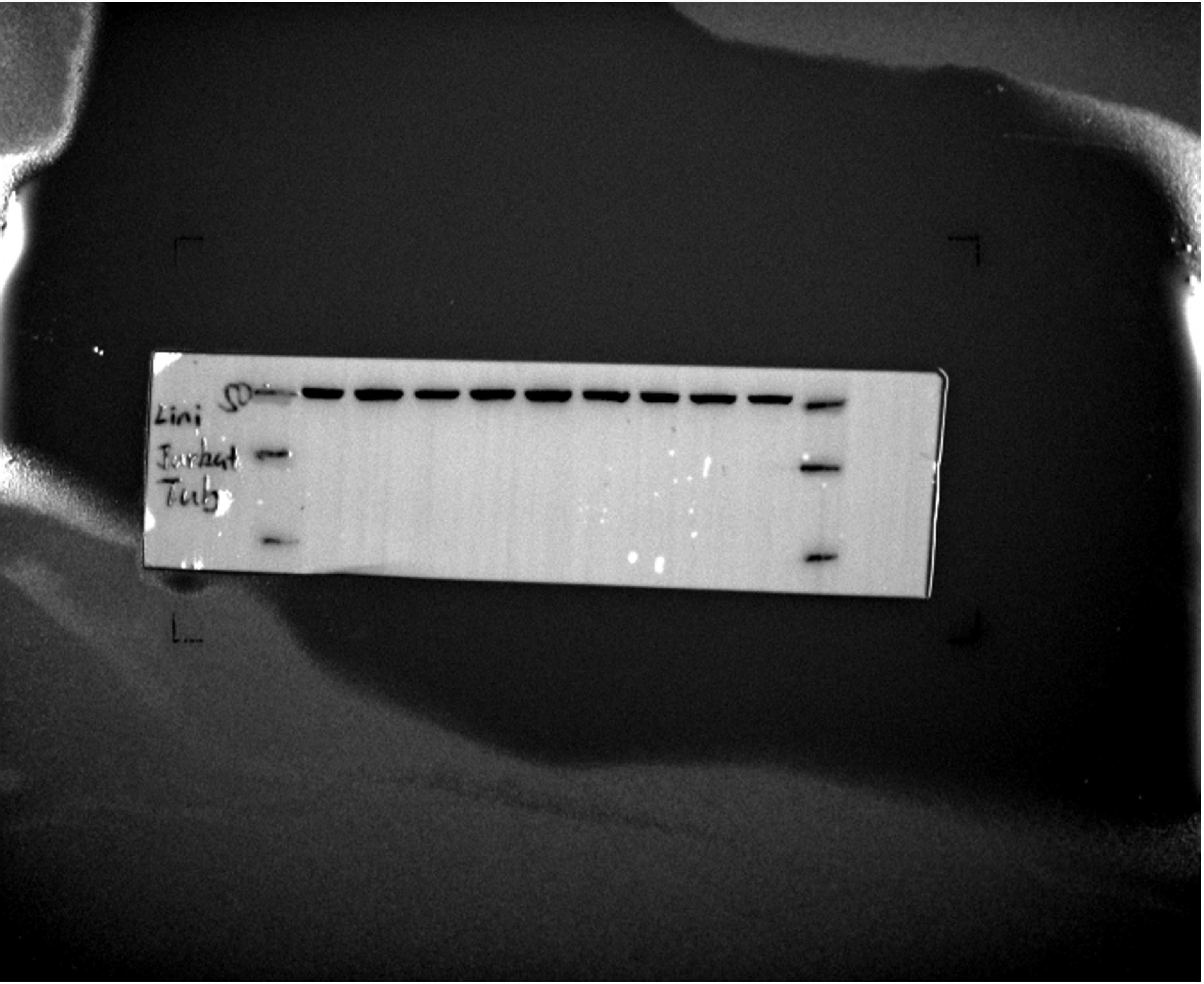

Figure5A FADD-deficient Jurkat-5 Tubulin

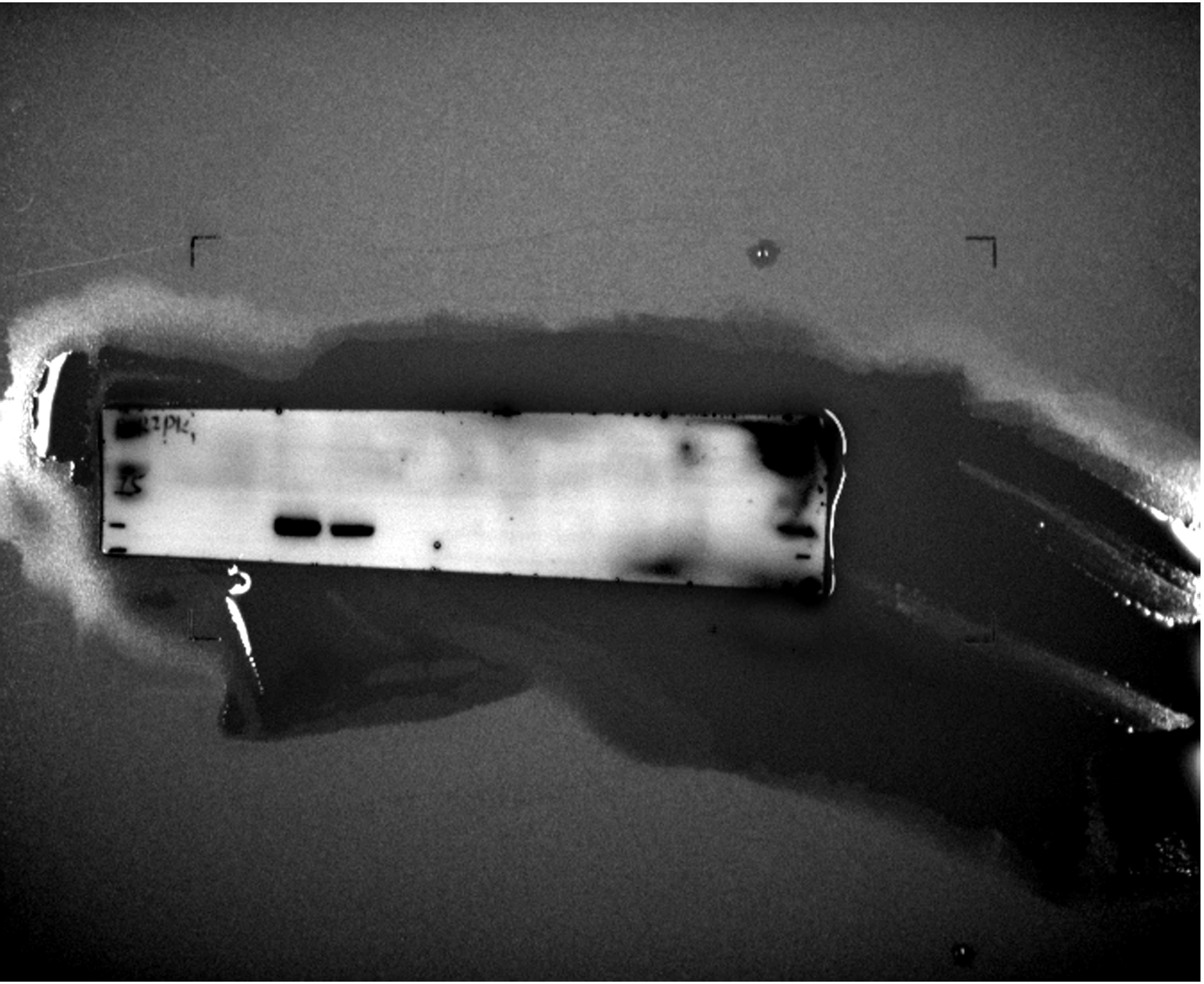

Figure5B MEFs-1 p-RIPK1(S166)

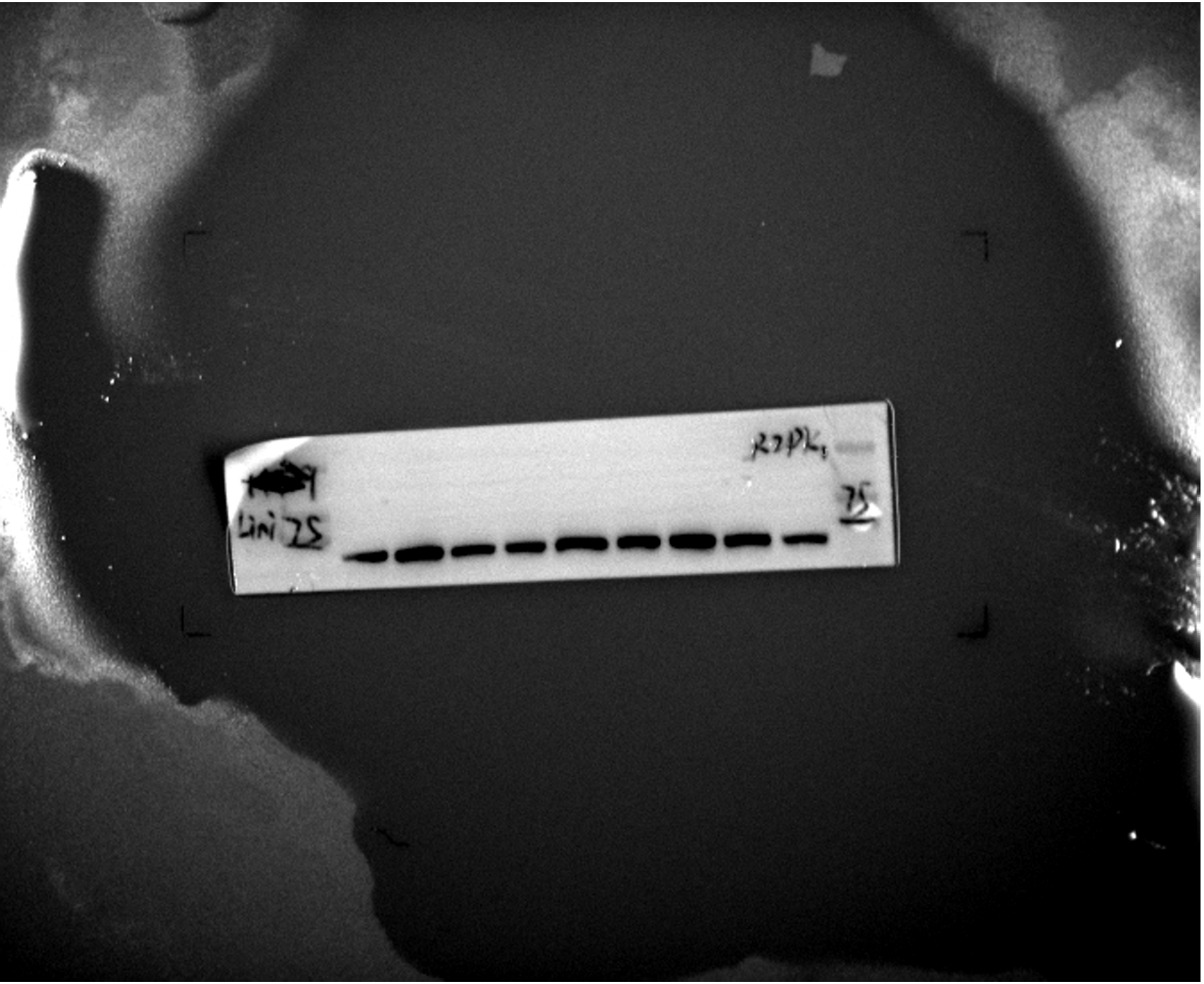

Figure5B MEFs-2 RIPK1

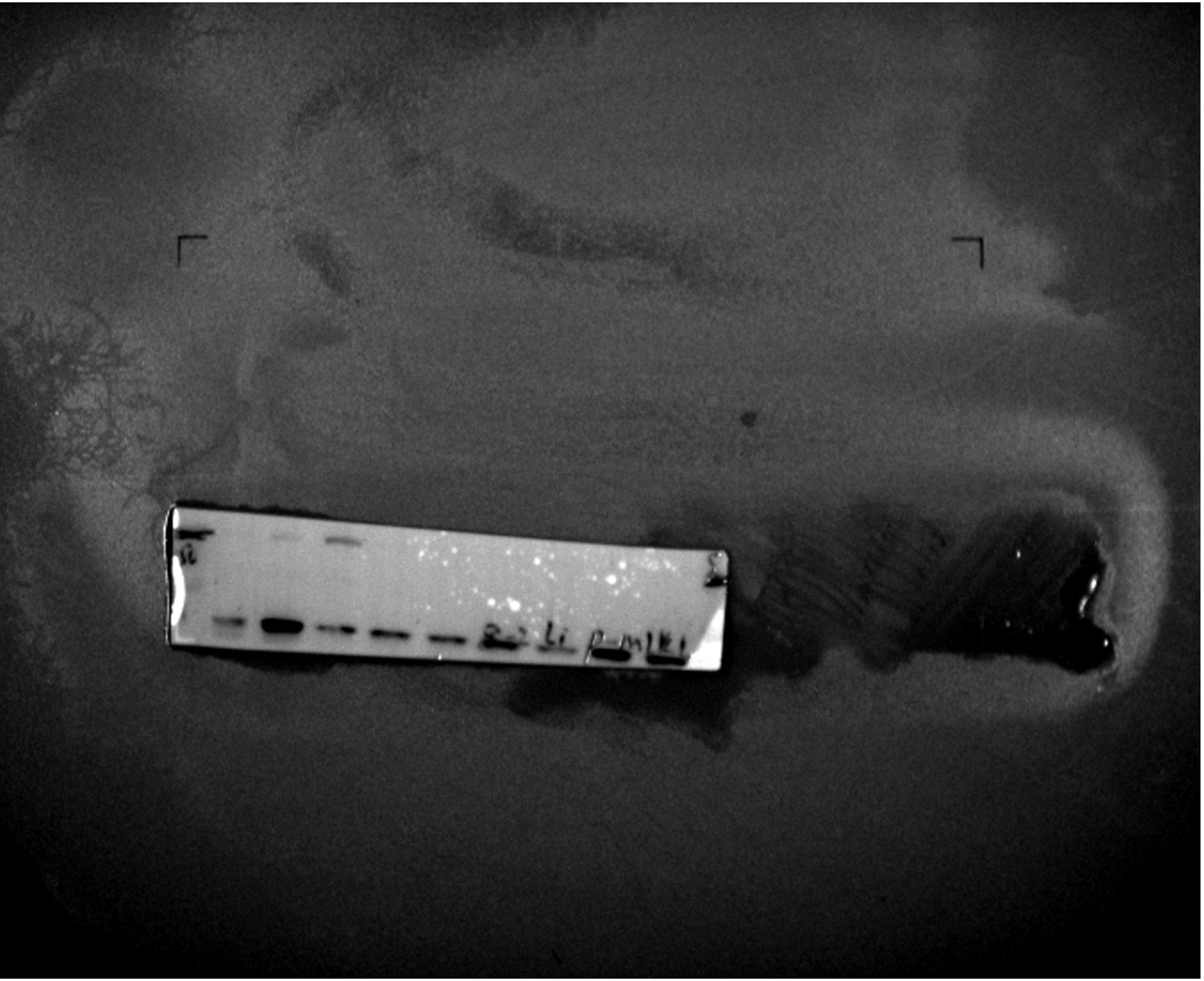

Figure5B MEFs-3 p-MLKL (S345)

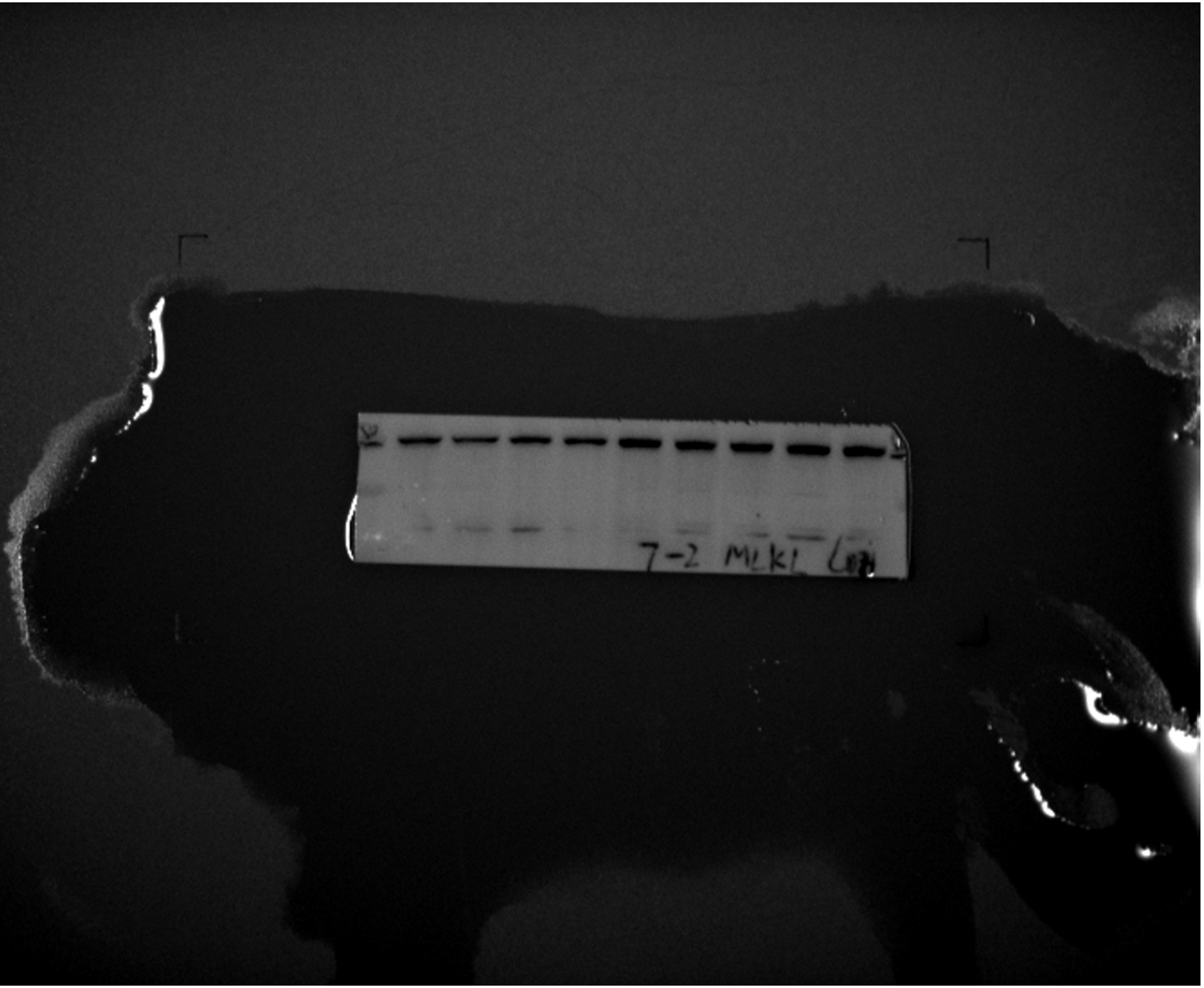

Figure5B MEFs-4 MLKL

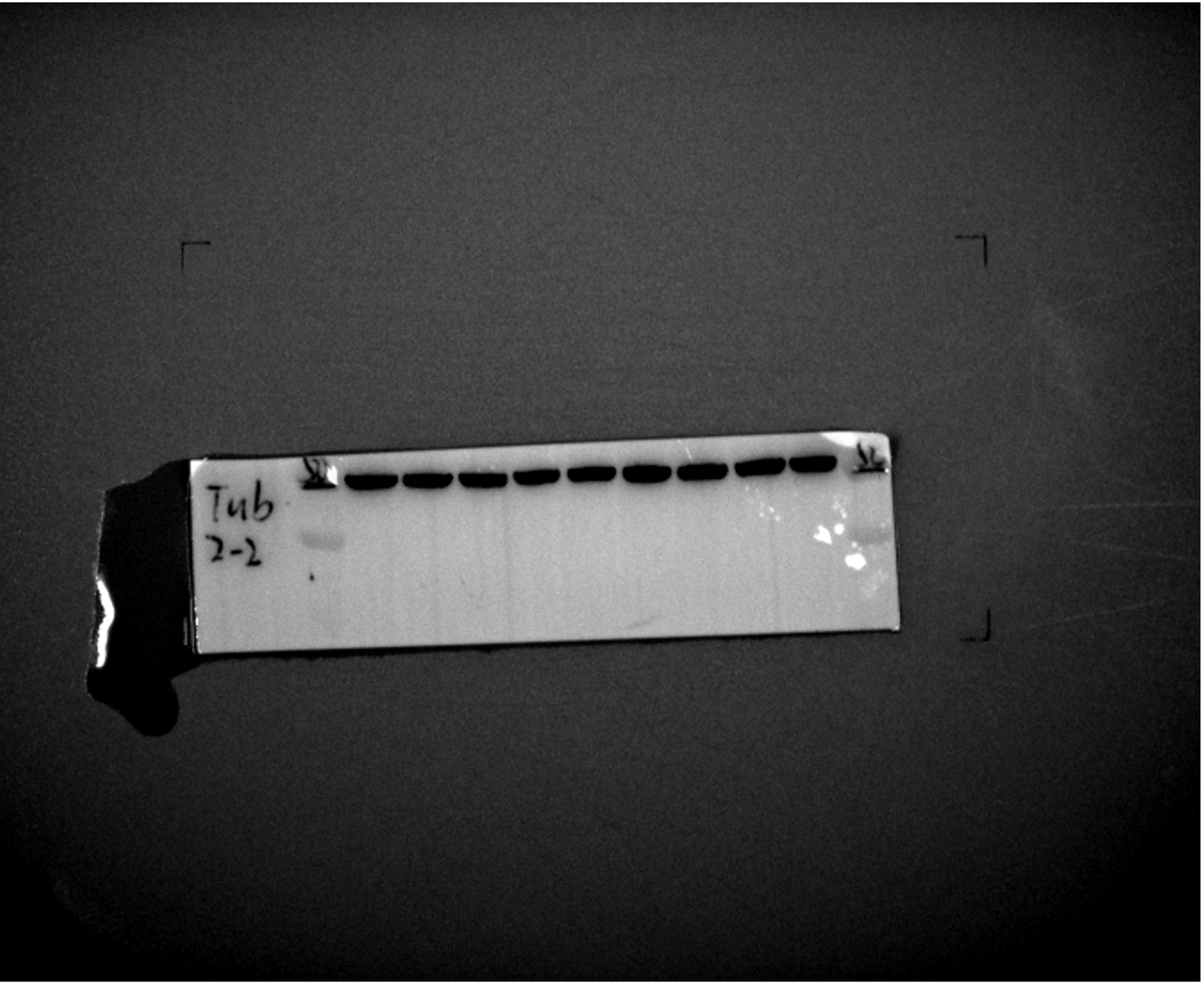

Figure5B MEFs-5 Tubulin

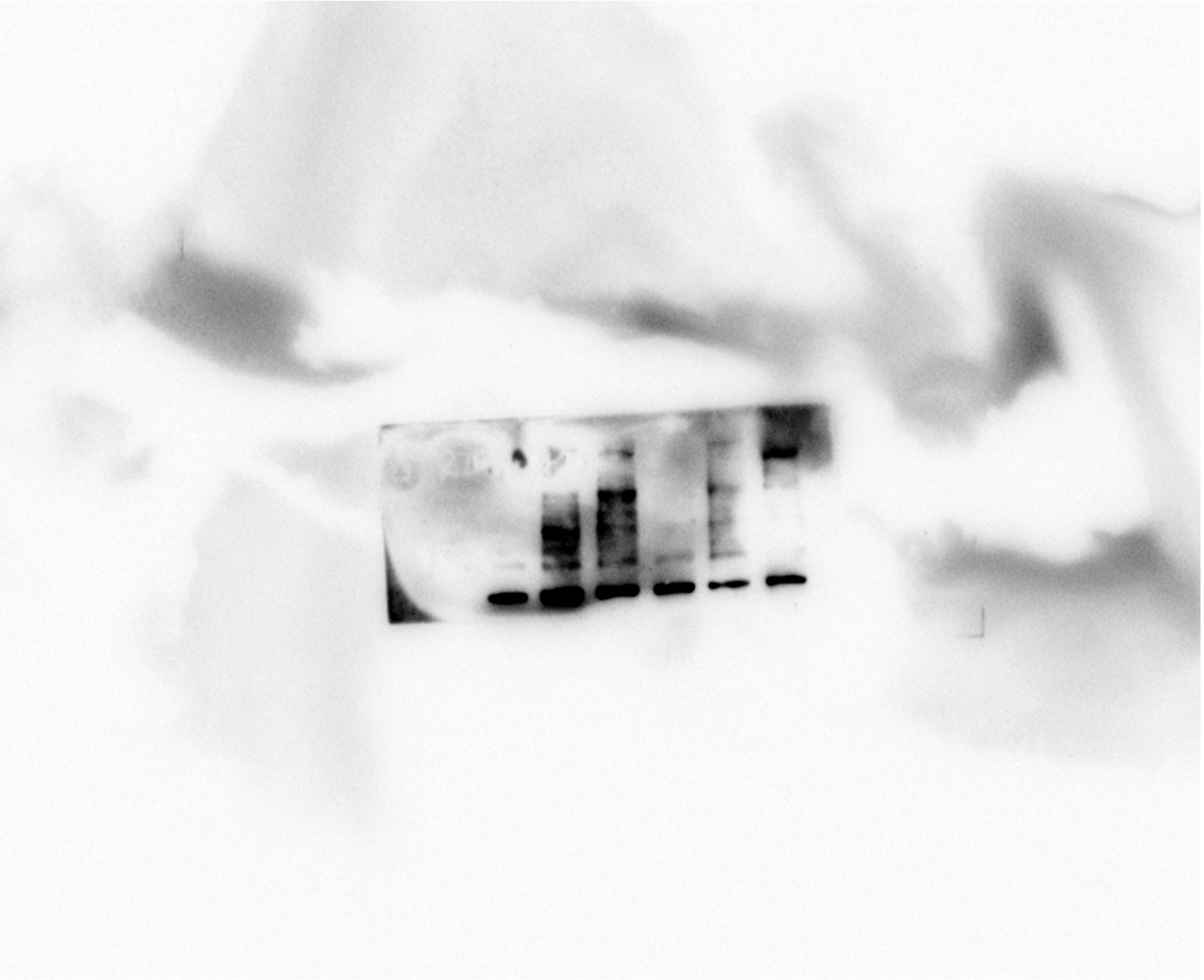

Figure5C MEFs-IP-1 Flag-RIPK1

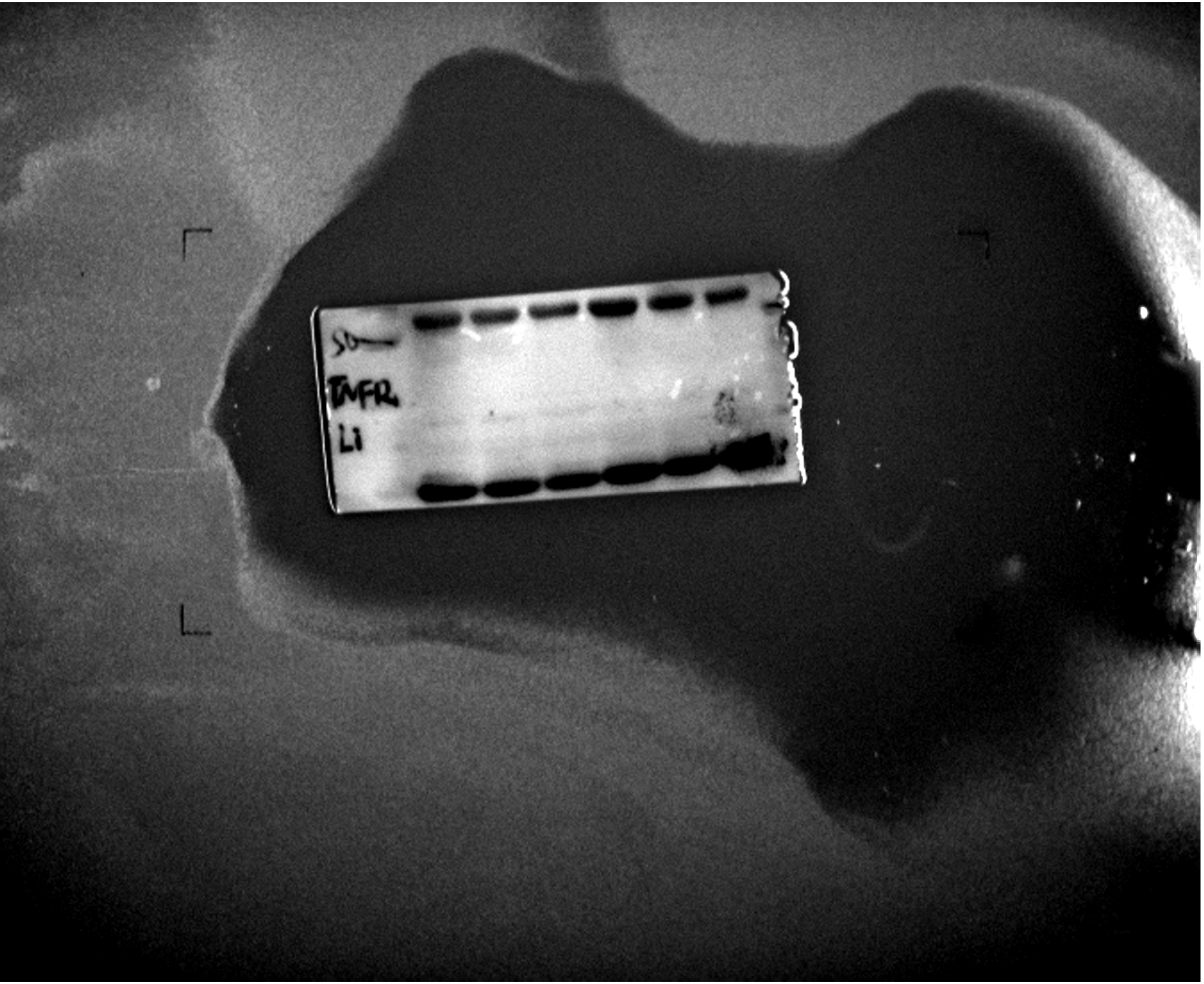

Figure5C MEFs-IP-2 TNFR1

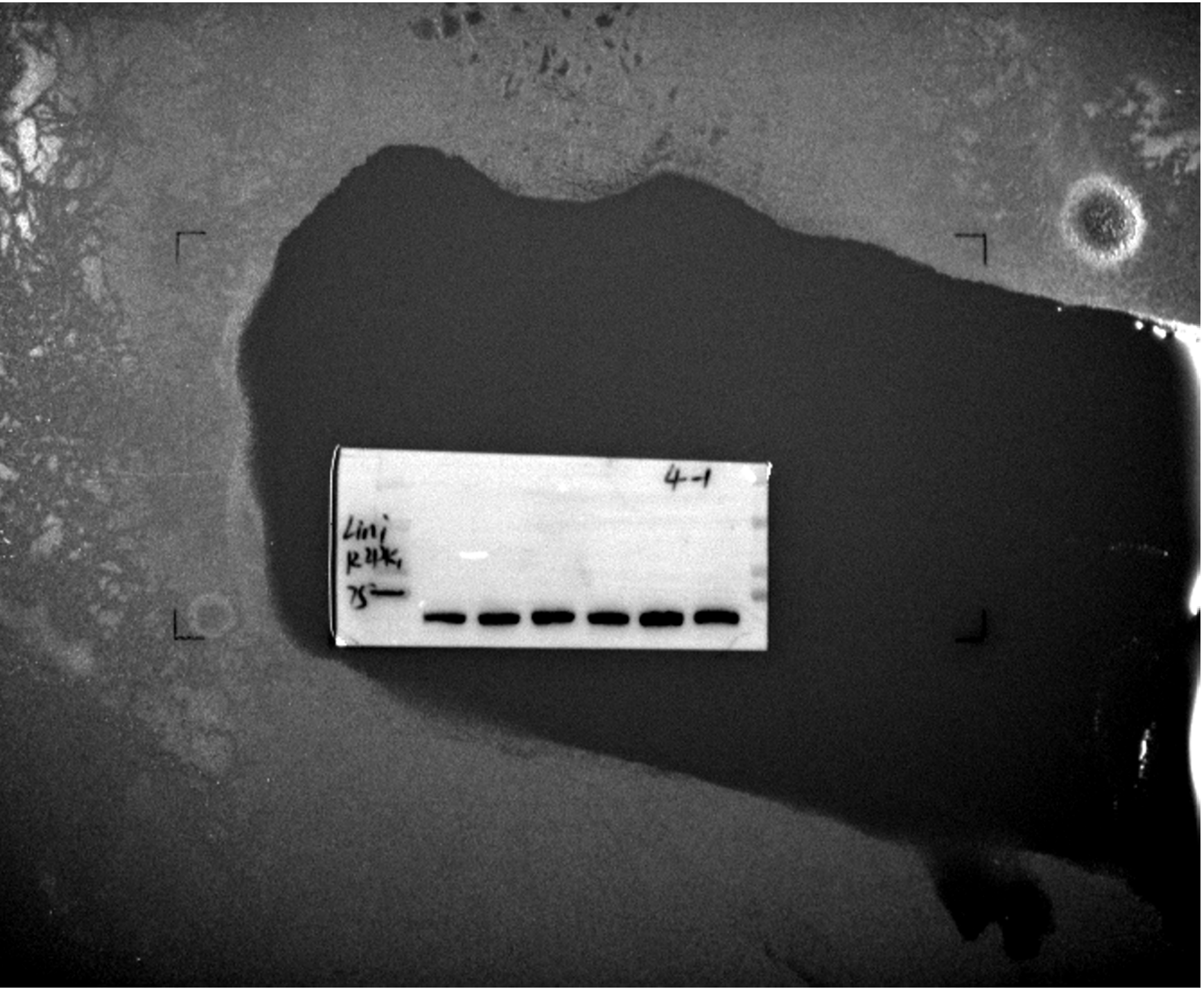

Figure5C MEFs-IP-3 RIPK1

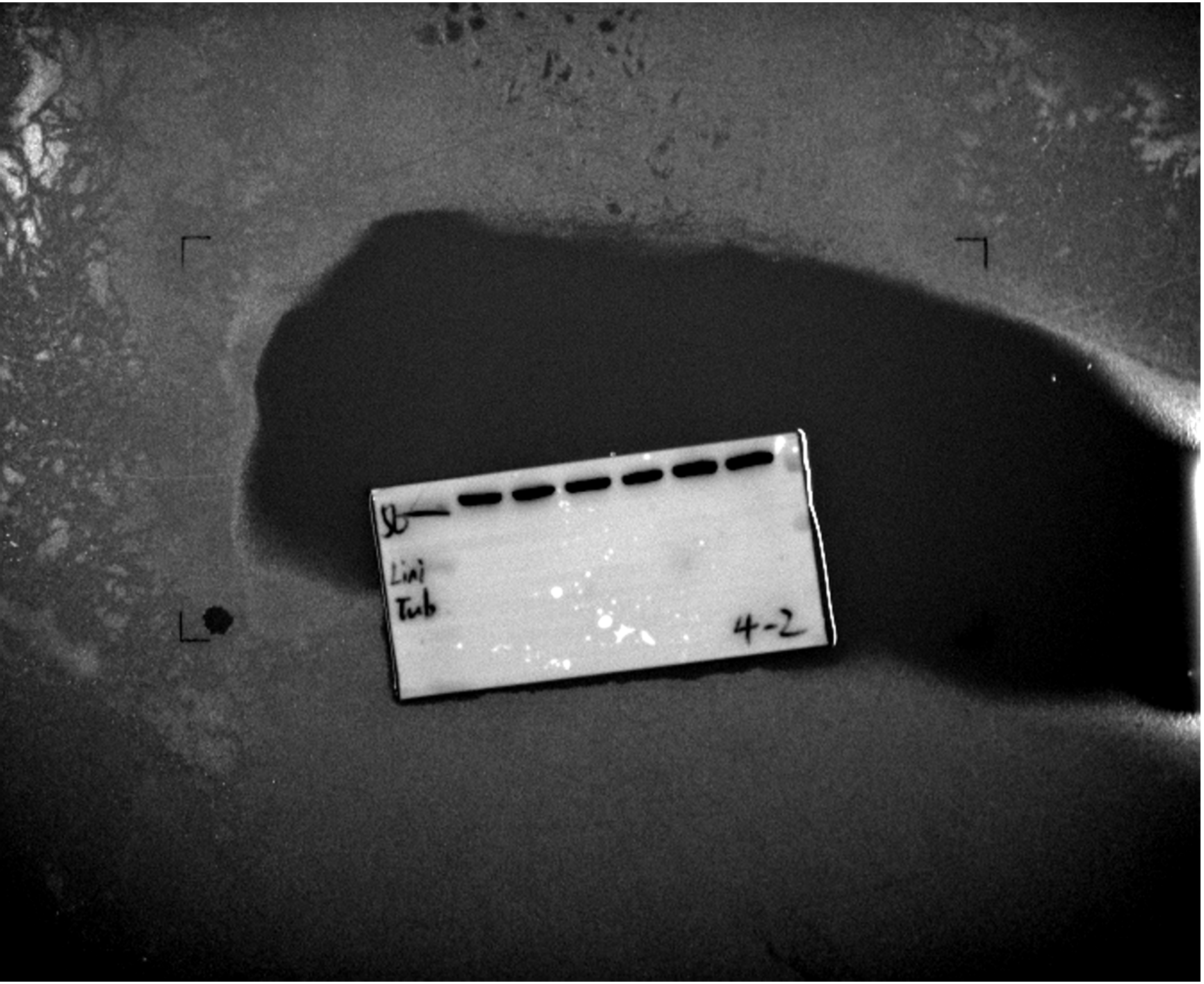

Figure5C MEFs-IP-4 Tubulin
