# Supplementary material for: Repositioning linifanib as a potent anti-necroptosis agent for sepsis
Source: Cell Death Discov. 2023 Feb 10;9:57. doi: 10.1038/s41420-023-01351-y (PMC9913023; doi:10.1038/s41420-023-01351-y)
Supplement: Supplementary file 11 — Ethics Statement [file 41420_2023_1351_MOESM11_ESM.pdf]

三峡大学实验动物福利与伦理审查表

CHINA THREE GORGES UNIVERSITY

THE TAB OF ANIMAL EXPERIMENTAL ETHICAL INSPECTION

编号（NO）：2019060B

一、项目与人员信息（Information of program and personnel）			
课题名称<br>Program	Linifanib通过抑制程序性坏死治疗全身炎症反应综合症的机制研究		
课题负责人<br>Name of principal Investigator	查运红		
单位<br>Department	三峡大学人民医院	动物实验负责人<br>Contact Person	何小燕
动物实验电 话<br>负责人<br>Contact PersonTel. No	15272198670	信箱<br>E-mail	hexiaoyan0721@163.com

参与动物实验操作人员姓名 Name

姓 名<br>Name	电话<br>Tel. No	信箱<br>E-mail
牟彦	18727727578	1207799792@qq.com
祝国锋	18186535398	2762159956@qq.com
李敏	15872530467	minli2018@ctgu.edu.cn
王佳	18871738660	1259841551@qq.com
尤晓玲	18871729919	545443594@qq.com
肖鑫	15727219621	1436128659@qq.com
顾谭蓉	18871730569	1207398552@qq.com

二、实验动物信息（Information of experimental animal）			
动物来源<br>Animal origin	三峡大学实验动物中心	品种/品系<br>breed/strain	SCXK（鄂）2017-0012<br>C57BL/6
数量（只）<br>Number (♀；♂)	80♂	动物级别<br>Grade	SPF
周/月龄<br>W/M Age	10-12周	体重（g）<br>Weight	

拟实验时间： 2019年7月-2021年7月

Experimental period

三、研究项目信息（Information）

1. 研究方法 （Research method）：

a. 观察 Linifanib 能否减轻 SIRS 模型小鼠的表型

b. 观察 Linifanib 对 SIRS 模型小鼠重要脏器损伤的影响。

2. 使用动物的必要性、合理性（The necessity and rationality of using animals）：

a. 为达到上述实验目的，目前未发现更好的替代此动物实验的方法。

b. 所用的动物品系和数量经过了精心设计和优化，尽量减少了动物的使用量，整个动物实验设计是必要和合理的。

3. 实验过程动物的福利（The welfare of animals during the experiment）：

a. 提供实验动物合适的居住环境，每笼不超过 5 只；

b. 提供灭菌过的饮水和全价的饲料，每周更换饮用水和饲料两次；

c. 专人负责实验动物的饲养管理，每天观察一次实验动物；

d. 凡采样或对实验动物造成疼痛的行为先实施麻醉 。

4. 动物实验项目的动物实验方案（Experimental Procedure）

a. 观察 Linifanib 能否减轻 SIRS 模型小鼠的表型。

实验动物：10-12 周 C57BL/6J 雄性小鼠每组 8 只

给药方式：灌胃

给药时间：注射 TNFα前 30 min 给药

给药剂量（分组）：对照组、模型组、Linifanib 组（50 mg/kg）、Nec-1 组（30mg/kg）

检测指标：1、肛温：10 小时内每 2 小时检测小鼠肛温，之后每 24 小时检测小鼠体温。

2、记录所有小鼠生存时间。

b. 观察 Linifanib 对 SIRS 模型小鼠重要脏器损伤的影响。

实验动物：10-12 周 C57BL/6J 雄性小鼠每组 12 只

给药方式：灌胃

给药时间：注射 TNFα前 30 min 给药

给药剂量（分组）：对照组、模型组、Linifanib 组（50 mg/kg）、Nec-1 组（30mg/kg）

检测指标：其中 4 只注射注射 TNFα后 6 小时灌注固定，HE 染色观察小鼠肝脏、肺脏、肾脏的形态学变化。其中 6 只注射 TNFα后 6 小时取新鲜组织，利用 RT-PCR 的方法检测各个脏器 IL-6 的表达水平。

5. 是否使用有毒（害）物质 （感染、放射、化学毒、其他）：
（Poisonous (harmful) material(infection, radiate, chemical poison and other)being used）：

无

6. 实验动物的死亡处理（Disposition of animals）：

深度麻醉动物后 CO_2 处死，动物尸体用塑料袋密封好后统一放置于实验动物中心的-20℃冰箱，由专业危费公司无害化处理。

四、审查依据（Inspection contents）

1. 该项目是否必须用实验动物进行实验，即能否用计算机模拟、细胞培养等非生命方法替代动物或用低等动物替代高等动物进行实验？<br>2. 表中所填申请人资格和所用动物的品种品系、质量等级、规格是否合适，能否通过改良设计方案或用高质量的动物来减少所用动物的数量？<br>3. 能否通过改进实验方法、调整实验观测指标、改良处死动物的方法，来优化实验方案、善待动物？
**声明：**<br>1. 我将自觉遵守实验动物福利伦理相关法规和各项规定，同意接受伦理委员会和实验动物室管理者的监督与检查；<br>2. 本人保证本申请表中所填内容真实、详尽和易懂。<br>**Declaration:**<br>1. I will abide by the law and regulation stipulation and accept the supervision and inspection by the committee and laboratory animal department.<br>2. The information I have given is accurate, detailed and comprehensive.<br>声明人：课题负责人签（章）<br>Declarant：Signature (stamp) of PI<br>动物实验负责人签（章）<br>Signature (stamp) of Director of animal experiment<br>年　月　日
福利伦理委员会审批意见<br>Approval opinion<br>☑ 批准 Approval　　☐ 不批准 Not approval<br>指定负责人签（章）：<br>Authorized Personnel Signature（Stamp）<br>年　月　日
